# Droplet removal by capillary lifting

**DOI:** 10.1038/s42005-026-02714-4

**Published:** 2026-06-12

**Authors:** Ke Sun, Jack R. Panter, Alvin C. M. Shek, Yonas Gizaw, Kislon Voïtchovsky, Halim Kusumaatmaja

**Affiliations:** 1 Department of Physics, Durham University, Durham, UK; 2 School of Engineering, Mathematics and Physics, University of East Anglia, Norwich, UK; 3 Whistler Center for Carbohydrate Research, Purdue University, West Lafayette, IN USA; 4 GreenChemSolutions LLC, West Chester, OH USA; 5 Institute for Multiscale Thermofluids, School of Engineering, The University of Edinburgh, Edinburgh, UK

**Keywords:** Fluid dynamics, Applied physics, Surfaces, interfaces and thin films

## Abstract

The removal of liquid droplets from solid surfaces is central to cleaning, coatings and oil recovery. While dewetting in binary fluid systems has been widely studied, the mechanisms governing dewetting in ternary systems involving an immiscible working liquid remain poorly understood. Here we show that a rising working liquid can capillary lift droplets into bridges extending from the solid surface to the air interface, leading to full, partial, or no droplet removal. Our theoretical model predicts, and experiments confirm, that the effectiveness of droplet removal can be tuned by manipulating the droplet contact angle with the solid and the interfacial tensions at play in the system. Significantly, dewetting can be enhanced by employing working liquids with high interfacial tension, in contrast to common surface cleaning strategies where surfactants are used to reduce interfacial tension. Our findings suggest new avenues for droplet manipulation, especially if concerned with reduced resources and a more sustainable environmental impact.

## Abstract (web summary)

Efficient removal of liquid droplets from solid surfaces is crucial for applications in cleaning, coating, and oil recovery. Here, the authors show that immiscible working liquids can capillary lift droplets into bridges whose dewetting efficiency can be tuned via droplet contact angles and interfacial tensions, offering a sustainable alternative to surfactant-based methods and opening new possibilities for resource-efficient droplet manipulation.

## Introduction

Droplet dewetting refers to the process where a liquid droplet retracts its contact from a solid surface. The phenomenon is central to a wide range of engineering and industrial applications, including self-cleaning surfaces^1^, inkjet printing^2^, and surface patterning^3,4^. To date, most studies of droplet dewetting focus on binary fluid systems, whereby the droplet is surrounded by air or an immiscible liquid. In such cases, a large body of literature has shown that liquid retraction can be driven by mechanisms, such as the droplet interfacial energy in the case of droplet impact^5^, spinodal dewetting^6^, loss of volume in the case of evaporation^7^ or change of effective contact angle due to external fields^8,9^.

In contrast, significant gaps remain in our understanding of dewetting in ternary systems where three fluid components are present together with the solid. Yet ternary dewetting is arguably more common than its binary counterpart, observed when a working liquid is used to induce droplet retraction, including for advanced oil recovery (immiscible water-alternating-gas displacement process)^10^, surface coatings^4^, hair dyeing^11^ and various forms of surface cleaning^1,12–14^. In the context of surface cleaning, there are strong economic and environmental pressures to devise new ways to displace soils (liquids or other forms of contaminants) from surfaces using less water, energy and surfactants^15–17^. This is further compounded by the emerging global water scarcity^18^ and the toxicity and low degradability of synthetic surfactants^19,20^.

In this study, we explore a fundamental mechanism of ternary dewetting (involving the droplet, the working liquid, and the gas phase) that we term capillary lifting, where a ‘soil’ droplet surrounded by air is lifted from the surface of a solid by a working liquid. Through a combination of experiments and simulations, we demonstrate the efficiency of capillary lifting across a wide range of liquids and substrates. Significantly, our results show that dewetting is favoured when the working liquid has a high interfacial tension, enabling the capillary force to effectively remove the droplet from the surface of the solid. This is different to conventional cleaning methods that employ surfactants to reduce the interfacial tensions so as to help solubilise the soil into the working liquid. We then propose a simple yet predictive model that describes the transition between dewetting and non-dewetting. Building on this, we extend our capillary lifting study to demonstrate its applicability in systems closer to real life applications: formulated working liquid and chemically heterogeneous soils. Our findings confirm capillary lifting as an effective droplet dewetting method that requires minimal resources (energy, water, surfactant) input, offering a more sustainable approach for droplet manipulation in ternary systems where the amount of working liquid is limited.

## Results and discussion

### Example of capillary lifting: dewetting and non-dewetting cases

**Fig. 1:**
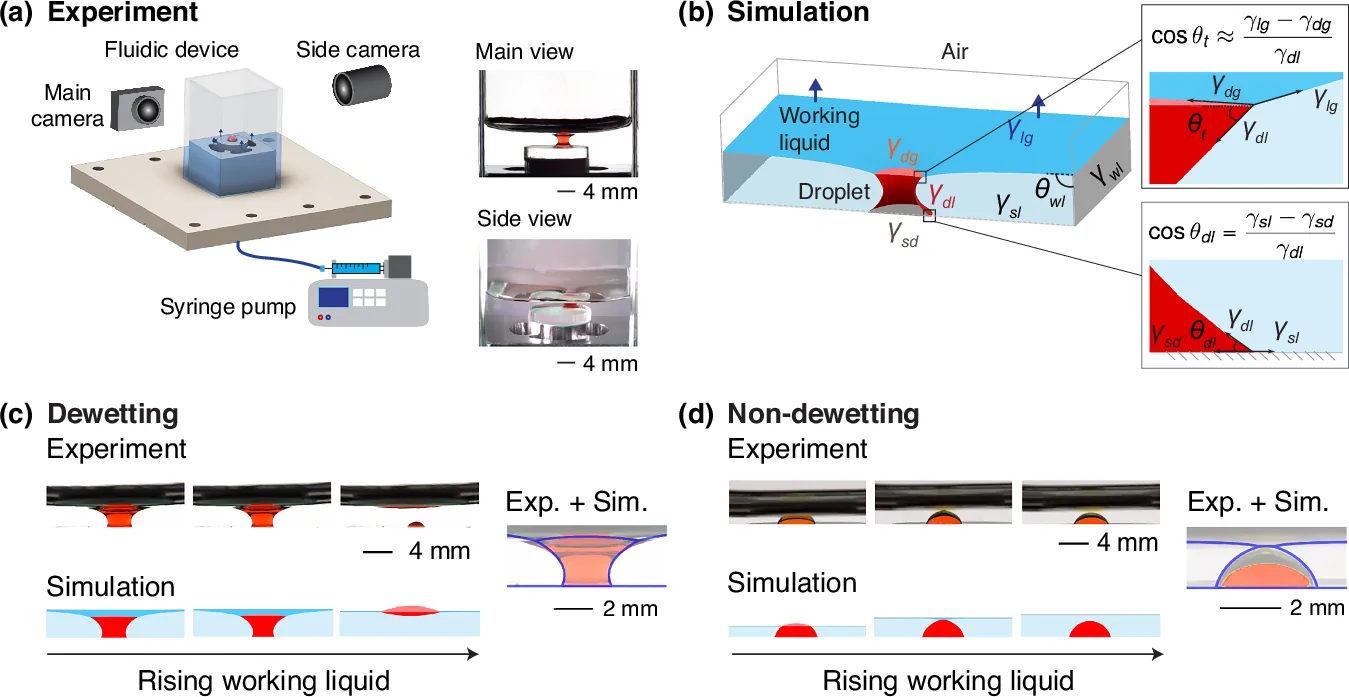
Capillary lifting of a droplet from a surface by a rising working liquid. **a** Experimental setup consisting of a dewetting device, a syringe pump, and a dual-camera imaging system. In the dewetting device, the central pillar supports the soil droplet, with the working fluid is injected from below by a syringe pump. The main camera records the droplet profile as its geometry evolves during removal, whereas the auxiliary side camera is positioned slightly above the substrate plane to visualise the droplet–air (and liquid–air) interface. **b** Simulation schematic diagram of a soil droplet being lifted from a solid substrate by a rising working liquid. The relevant thermodynamics parameters are the interfacial tensions (or energies) of the solid substrate–droplet *γ*_sd_ (grey), droplet–gas *γ*_dg_ (light red), liquid–gas *γ*_lg_ (blue), substrate–liquid *γ*_sl_ (hidden beneath liquid), droplet–liquid *γ*_dl_ (red), liquid–wall *γ*_lw_ (grey), and wall–gas *γ*_wg_ (transparent). The receding contact angle of the droplet in liquid is *θ*_dl_ and the effective top contact angle is *θ*_t_, which can be expressed in terms of the balance of interfacial tensions (see insets). **c**, **d** Examples of capillary lifting observed experimentally and in simulations for two typical situations. For the combined simulation and experimental panels, the simulated profiles are shown as blue lines atop the experimental results. **c** Successful dewetting of a dyed tetradecane droplet on a poly(methyl methacrylate) (PMMA) surface by water. **d** Failed dewetting of a dyed water droplet on PMMA by tetradecane. The dyed water droplet is colourised red for visual consistency (naturally yellow). Scale bars in (**a**), (**c**), and (**d**): 4 mm for the experimental images and 2 mm for the experimental and simulation overlays in (**c**) and (**d**).

**Fig. 2:**
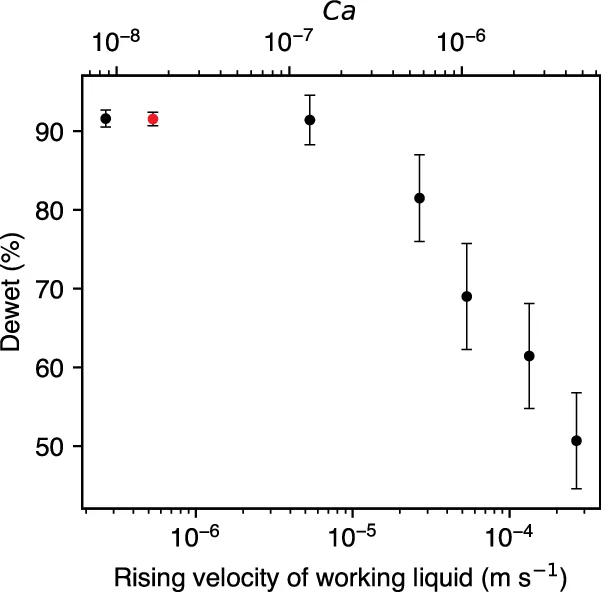
Capillary lifting at different rising velocities of the working liquid. A tetradecane droplet on a poly(methyl methacrylate) (PMMA) substrate is lifted by injecting water at velocities ranging from 10^−7^ to 10^−4^ m s^−1^. The dewetting outcome changes from nearly complete detachment at lower velocities to partial detachment at the higher velocities. The data point highlighted in red corresponds to the dewetting velocity employed in this work (unless specified otherwise). These velocities correspond to capillary numbers in the range ${Ca} \sim {10}^{-9}-{10}^{-6}$. Error bars represent standard deviations.

To investigate capillary lifting in ternary systems, we employed an integrated experimental–simulation approach. Details of the experimental procedure can be found in Methods. In brief, we designed a fluidic device (Fig. 1a) in which a droplet ( ~ 10 µL) is deposited on a central substrate. The working fluid is injected from below through four symmetrically arranged holes using a syringe pump, ensuring uniform lateral distribution and a controlled rise velocity of ~5 × 10^−7^ m s^−1^, and allowing the system to operate near quasi-equilibrium (see details of kinetic investigation in Fig. 2 and associated text). As the working fluid rises, the droplet forms a capillary bridge connecting the bottom substrate and the top air–droplet interface, while being laterally surrounded by the working fluid. This resembles classical capillary bridges between parallel solid plates, except that here the air interface acts as a deformable soft surface; we therefore refer to this structure as a capillary bridge. The bridge geometry evolves during fluid injection and is recorded using a dual-camera system. The main camera captures the bridge shape, allowing direct comparison with simulation profiles, while the side camera provides a complementary perspective from a slightly elevated angle, clearly revealing the droplet–air interface (as shown in Fig. 1a and detailed in Supplementary Note 1). This experimental setup enables us to capture the progressive changes in the bridge’s shape as the working liquid rises, including the deformations and potential rupture, which ultimately lead to distinct dewetting outcomes.

The experiments are complemented by computer simulations (Fig. 1b) using Surface Evolver^21^. The simulation framework (see Methods for details) incorporates the relevant interfaces and liquid properties, such as interfacial tensions and fluid densities (measured parameters provided in Supplementary Note 2). The working fluid is added incrementally, and at each step the system is allowed to relax to an energy-minimised state. The configuration at each step is recorded and compared with the experimental observations, continuing until the final dewetting outcome is reached. Since the capillary length is comparable to the soil droplet size, gravitational force is included in our analysis (see Supplementary Note 3 for gravity impact) together with the relevant interfacial tensions. The total energy of the system can be expressed as:

1 $${E}_{{{\rm{Total}}}}=\mathop{\sum }\limits_{i,j}{\gamma }_{{ij}}{A}_{{ij}}+g\mathop{\sum }\limits_{k}{\int }_{{\!\!\!\!\!V}_{k}}{\rho }_{k}{zdV}$$

where *γ*_*ij*_ represents interfacial tensions between two fluids *i* and *j*, *A*_*ij*_ is the corresponding interfacial area, *g* is the gravitational acceleration, *z* is the vertical coordinate, *ρ*_*k*_ and *V*_*k*_ denote densities and volumes of the fluids involved in the system. For simplicity, we take a quasi-static approximation, corresponding to the limit of *Ca* → 0 (typically, ${Ca} \sim {10}^{-8}$ in our experiments and we do not observe significant dynamic effects for ${Ca}\lesssim {10}^{-7}$). In this limit, we slowly and incrementally increase the volume of the invading working liquid and carry out energy minimisation for each corresponding volume (see the Methods section for additional details of the numerical scheme).

Having established both the experimental and simulation frameworks, we next present typical dewetting configurations and outcomes. Figure 1c illustrates a situation where a liquid droplet (‘soil’, in red) is lifted from the solid by the working liquid. During the process, the droplet extends vertically through the working liquid to form a capillary bridge between the solid and the air. As the level of the working liquid rises, the bridge eventually ruptures, with most of the soil droplet removed from the solid. Alternatively, the soil droplet can remain bound to the surface (Fig. 1d). Before discussing the physics implications of the results shown in Fig. 1c–d, we note the excellent agreement between the experimental measurements and equilibrium computer simulations. This confirms the predictive power of simulations, provided the capillary rise occurs quasi-statically and thermodynamic equilibrium is well approximated at all times. Experimentally, this is not obvious despite the low rising speeds. We therefore decided to explore systematically the experimental window where these conditions are met (Fig. 2). For rising velocities below 5 × 10^−6^ *m s*^−1^ (capillary number ${Ca}\lesssim {1\times 10}^{-7}$), the dewetting percentage remains essentially unchanged, indicating that the system reaches a quasi-static equilibrium. For velocities above ${5\times 10}^{-6}m\;{s}^{-1}({Ca}\gtrsim {1\times 10}^{-6})$, the dewetting percentage decreases with increasing velocity, demonstrating that the system enters a kinetic regime. A similar transition from a thermodynamically dominated to a kinetic regime occurs in numerous capillary-driven phenomena where operation in the low-*Ca* regime ensures that capillary forces dominate over viscous forces, enabling precise control over interfacial dynamics. For example, quasi-static operation is used for the peeling of hydrophobic films^22^, meniscus-guided coating of organic electronics^23^, aggregation of elastic objects^24^, and complex colloidal self-assembly^25^. Accordingly, we maintained quasi-static experimental measurements throughout this study (data point highlighted in red in Fig. 2).

Our results in Fig. 1c–d show that water can remove >90% of a tetradecane droplet, but tetradecane cannot displace a water droplet. This observation may seem surprising since tetradecane has clearly the strongest affinity for poly(methyl methacrylate) (PMMA): tetradecane exhibits near-complete wetting on PMMA in air (contact angle ~ 0°) while water droplets show a significantly higher contact angle of 71.2 ± 3.9° (see Supplementary Note 4 for contact angle measurements). The key to understand this apparent contradiction is that effective capillary lifting requires a high interfacial tension for the working liquid. Here, water has a significantly higher interfacial tension (72 mN m^−1^) than tetradecane (26.6 mN m^−1^). Thus, for such ternary dewetting, the use of surfactants and other small molecules to reduce interfacial tension (explored later in the paper), ubiquitous in conventional surface cleaning strategies, can actually degrade performance. The fact that capillary lifting mechanism benefits from the absence of surfactants opens an effective avenue for an alternative droplet removal and manipulation approach that is more sustainable and environmentally friendly. However, given that three fluids are involved, the space parameter of capillary lifting is considerable. First, it is not clear a priori what are the key parameters controlling the lifting mechanism and its efficiency. Second, the principle of capillary lifting entails moving from a non-dewetting situation (Fig. 1d) to a dewetting (Fig. 1c) where lifting takes place. It is therefore crucial to understand how the transition between these two situations occurs when manipulating the relevant parameters. Third, capillary lifting needs to function with to real-world complex soils (chemically heterogeneous) if to offer a meaningful alternative to traditional surfactant-based methods.

### Dewetting regime diagrams

**Fig. 3:**
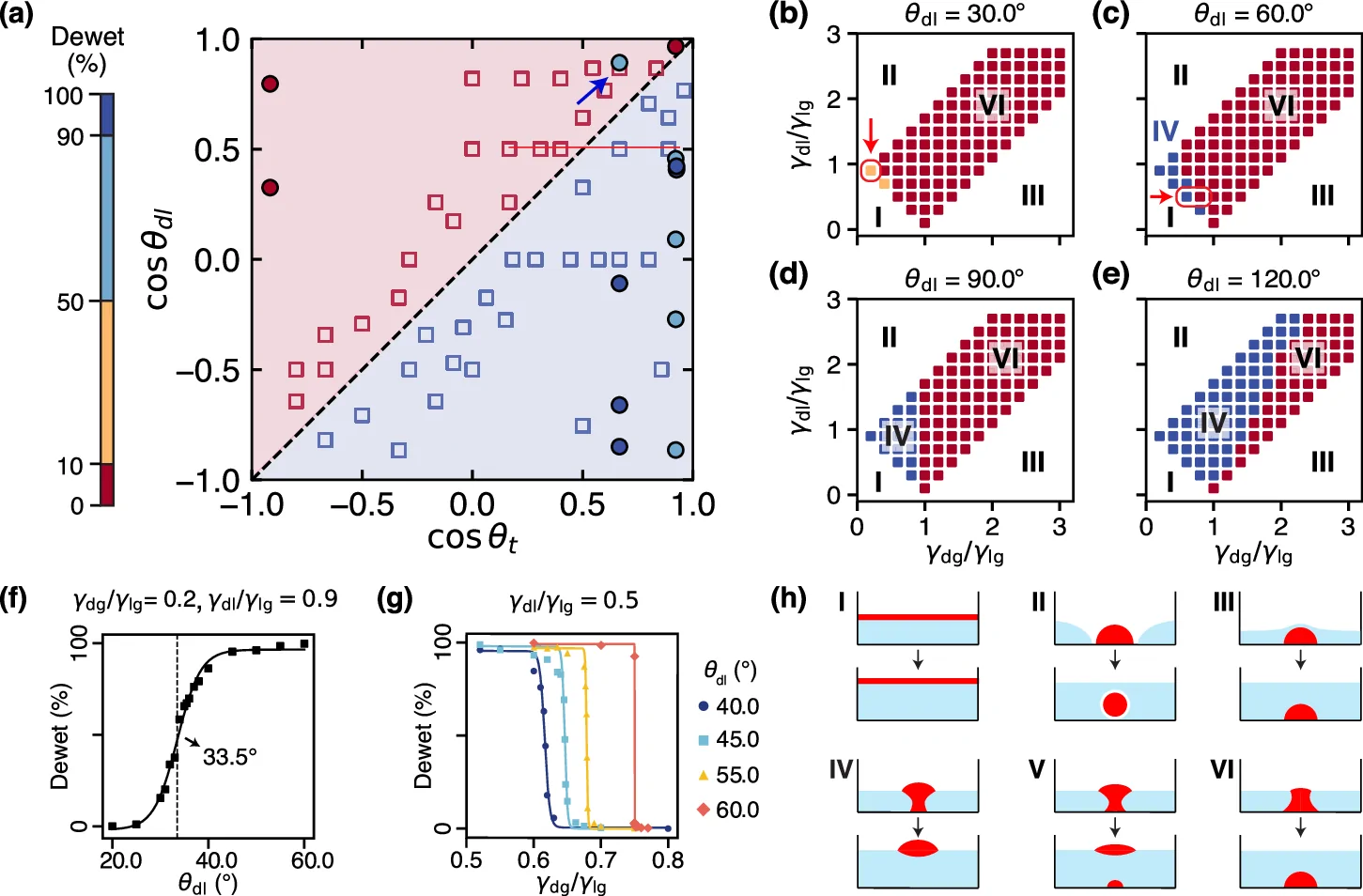
Experimental and simulated dewetting regime diagrams across various interfacial systems. **a** Ternary dewetting simulation (open squares) and experiments (filled circles) shown as a function of the effective top contact angle (*θ*_t_) and the receding contact angle of the droplet in liquid (*θ*_dl_). The background colour indicates the theoretical prediction for dewetting (cos *θ*_t_ > cos *θ*_dl_, light blue shaded region) and non-dewetting (cos *θ*_t_ < cos *θ*_dl_, light red shaded region). The colour of the experimental and simulated data points represents the measured degree of dewetting, as indicated by the colour bar. **b**–**e** Simulated dewetting phase diagrams across a range of *θ*_dl_ and interfacial tensions *γ*_dg_ (droplet–gas), *γ*_dl_ (droplet–liquid), and *γ*_lg_ (liquid–gas). Blue squares indicate dewetting, whereas red squares indicate non-dewetting, consistent with the colour scheme in (**a**). **f** Dewetting behaviour for an interfacial system (*γ*_dg_/*γ*_lg_ = 0.2, *γ*_dl_/*γ*_lg_ = 0.9, red arrow in (**b**)) as a function of *θ*_dl_. The black dashed vertical line indicates the midpoint between dewetting and non-dewetting, corresponding to ~50% dewetting at *θ*_dl_ = 33.5°. **g** Transition boundary from dewetting to non-dewetting at *γ*_dl_/*γ*_lg_ = 0.5 with varying *γ*_dg_/*γ*_lg_. Results for different *θ*_dl_ are shown, including for *θ*_dl_ = 60.0° (red arrow in (**c**)). **h** Schematics of the six droplet morphology regimes of (b–e), showing the droplets in red, the working fluid in blue, and the air in white.

Using computer simulations, we systematically explored wide-ranging physical parameters (Fig. 3), developing regime diagrams for capillary lifting. Experiments scattered across the diagram validate the predictions. The experimentally investigated soil droplets range from pure liquids such as tetradecane and water to real-world contaminants, including squalane (used in cosmetics and base oil lubricant), olive oil, and used engine oil. For the working fluid, we used either pure liquids or low-concentration glycol ether formulation relevant to industrial applications (0.45 wt% tripropylene glycol methyl ether, 0.45 wt% diethylene glycol hexyl ether and 0.45 wt% ethylene glycol hexyl ether in water). We tested capillary lifting on commercial substrates with varying surface energies, including polytetrafluoroethylene (PTFE), polycarbonate, PMMA, glass, and stainless steel. The liquid interfacial tensions and wetting properties were experimentally characterised using pendant and sessile drop setups, as detailed in the Methods section and the Supplementary Notes 2 and 5. The contact angle hysteresis of each tested dewetting system, together with the corresponding surface topography and roughness characterisation, is shown in supplementary section Supplementary Note 6.

**Fig. 4:**
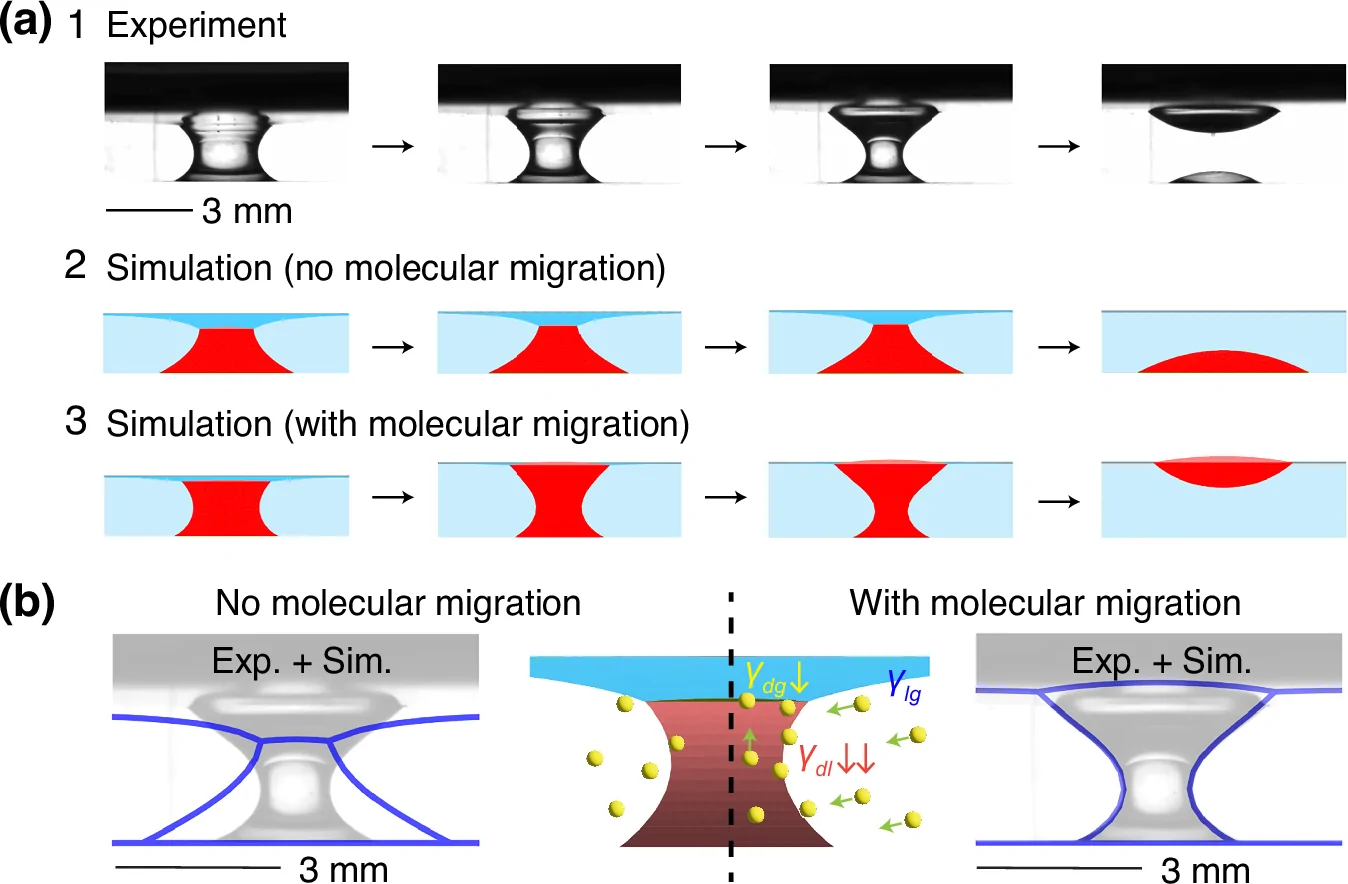
Capillary lifting mechanism in complex systems where droplets or working fluids are not chemically pure. **a** Dewetting using a low-concentration formulation as the working liquid. Simulations using binary interfacial tensions fail to capture the experimental profile. In contrast, incorporating the impact of molecular migration between interfaces yields good agreement with experiments (see text). **b** Experimental snapshots overlaid with simulation profiles (blue lines), shown with (right) and without (left) molecular migration. A schematic illustrates the mechanism in the middle. All scale bars represent 3 mm.

From Fig. 3a, it is clear that the dewetting and non-dewetting regions follow a trend that can be described theoretically as a function of the receding contact angle of the droplet in liquid, *θ*_dl_, and the effective top contact angle, *θ*_t_, which is determined by the balance of interfacial tensions (see Fig. 1b). This can be rationalised from simple geometrical considerations by analogy with capillary bridges. A bridge stability is determined by the contact angles on the top and bottom substrates, here by analogy *θ*_dl_ and *θ*_t_. Dewetting is favoured when $\cos {\theta }_{{dl}} < \cos {\theta }_{t}$ (bottom right corner in Fig. 3a), as the bottom contact radius vanishes faster than the top contact radius^26,27^. This simple approximation yields an excellent agreement between experiments, simulations and theory, though small deviations are observed close to the boundary line since the air interfaces are slightly curved (see details in Supplementary Note 7). A clear outlier (blue arrow in Fig. 3a) occurred when a formulated solution is used as the working fluid instead of a pure liquid. This can be accounted by considering molecular migration and will be discussed in the next section and in Fig. 4.

All possible morphologies from the capillary lifting mechanism are shown in Fig. 3b–e, which capture regime diagrams in the space of interfacial tension ratios *γ*_dl_/*γ*_lg_ and *γ*_dg_/*γ*_lg_ for four different values of *θ*_dl_. For each diagram, six regions can be broadly defined, labelled I to VI and illustrated as cartoon in Fig. 3h. In regime I (*γ*_dg_ + *γ*_dl_ < *γ*_lg_), the soil droplet completely spreads, forming a film at the working liquid–gas interface. In II (*γ*_dg_ + *γ*_lg_ < *γ*_dl_), an anti-bubble forms whereby the soil droplet is cloaked by the air, which in turn is surrounded by the invading liquid. In III (*γ*_dl_ + *γ*_lg_ < *γ*_dg_), the droplet disfavours contact with the gas phase and always remains attached to the solid substrate. In IV, V and VI, the three fluid interfaces can form a Neumann triangle, and a capillary bridge is formed between the solid surface and the air interface. The droplet can dewet, as previously illustrated in Fig. 1c, and we can further distinguish whether we find full (IV) or partial (V) dewetting. In VI, the droplet does not dewet from the surface.

Importantly, we find that dewetting can occur for droplet contact angles significantly lower than 180°. Additionally, as *θ*_dl_ increases, the droplet is more likely to dewet across various interfacial tension ratios (Fig. 3b–e). This is expected since higher *θ*_dl_ indicates a lower interfacial energy between the working liquid and the surface, compared to the droplet. Dewetting is also preferred for lower *γ*_dg_ and higher *γ*_lg_, which effectively lower *θ*_t_, as they destabilise the submerged droplet and stabilise the liquid lens geometry at the air interface.

We now focus on the sharpness of the boundary between full and non dewetting as predicted by the computational model. An example of a partial dewetting simulation is provided in Fig. S6 (Supplementary Note 7). When all interfacial tensions are held fixed (arrow in Fig. 3b), the transition typically follows a smooth hyperbolic tangent profile when varying *θ*_*dl*_ (Fig. 3f). In contrast, when *θ*_*dl*_ is held constant, and the interfacial tension ratio *γ*_dg_/*γ*_lg_ varies (arrow in Fig. 3c), the transition becomes more abrupt and sharpens further with increasing *θ*_*dl*_ (Fig. 3g). These findings suggest that efficient dewetting can be achieved at relatively low contact angles by tuning interfacial tensions in a ternary system. For instance, abrupt dewetting transitions can be triggered by a slight decrease of *γ*_dg_/*γ*_lg_. We note that the partial dewetting states captured in the simulation model (Fig. 3f–g and Supplementary Note 7) occur only within a very narrow region of interfacial parameter space.

One important assumption of the model is that of perfectly smooth and homogeneous surfaces. However, experimentally most surfaces inevitably exhibit some roughness and chemical heterogeneities. As a result, we typically observe contact line pinning, which leads to residues remaining on the surface upon dewetting and a slight deviation in the percentage of removal between experiments and simulations (as shown in Fig. 1c and the experimental–numerical comparison in Supplementary Note 8). Consequently, partial dewetting is more prominent in experiments than in simulations.

### Towards capillary lifting in real-life applications

Another important aspect of surface cleaning is the common use of water-based formulations to enhance outcomes in binary systems. For comparison, we conducted capillary lifting experiments of tetradecane droplets from a PTFE substrate using the low-concentration glycol ether formulation (Fig. 4a). The interfacial tensions *γ*_lg_ and *γ*_dl_ were experimentally measured via the pendant drop (see the Methods section and Fig. S7a of Supplementary Note 9), which is commonly applied to binary fluid systems. However, unlike for the pure liquid systems, when these interfacial tensions are input into the model, the predicted outcomes diverge significantly from experimental results (see panels 1–2 in Fig. 4a). The associated dewetting prediction is the outlier data point highlighted in Fig. 3a, confirming that simple pairwise interfacial tension-based predictions for pure liquid no longer hold for complex ternary systems. The insufficiency of pairwise tensions suggests that additional mechanisms are at play.

To answer this discrepancy, we need to consider molecular migration across the ternary system. When a droplet forms a capillary bridge between the solid and the air interface, the simultaneous increase in liquid–droplet interfacial area and exposure to the hydrophobic air phase can stimulate amphiphile redistribution within a composite solution, here the formulation. This results in different molecular concentrations at the different interfaces. For example, it has been shown that oil vapor in the air phase can promote surfactant adsorption at water–air interfaces through cooperative interactions with oil molecules that thermodynamically stabilise the adsorption layers^28^. Here, the mechanisms and dynamics of surfactant migration are not known a priori. We start from the so-called Hansen solubility parameters^29^, which quantify the cohesive energy of a substance and help predict its solubility in another material. We find that the average Hansen distance of the glycol ethers (in the formulation) and the soil oils (tetradecane or decane) is ~2.6 times smaller than the distance to the water phase (see details of Hansen distance calculation in Supplementary Note 10). This indicates a strong preference of the glycol ether for the hydrophobic interfaces, therefore influencing the resulting interfacial tensions in the ternary system. The impact of this migration on the resulting interfacial tensions can be characterised with the Neumann triangle method^30^, by depositing an oil droplet on the liquid–air interface to mimic ternary dewetting conditions (see the Methods section and Fig. S7b of Supplementary Note 9). The results demonstrate a shift in the interfacial tensions from *γ*_dg_/*γ*_lg_ = 0.94 and *γ*_dl_/*γ*_lg_ = 0.64 in the binary system, to *γ*_dg_/*γ*_lg_ = 0.84 and *γ*_dl_/*γ*_lg_ = 0.24 in the ternary system, where liquids are exposed to air. This change confirms the importance of glycol ether migration despite their relatively low concentration (1.35 wt%). Incorporating the corrected interfacial tensions into our model yields a good agreement between experimental and computational results (see panels 1 and 3 in Fig. 4a, and b).

**Fig. 5:**
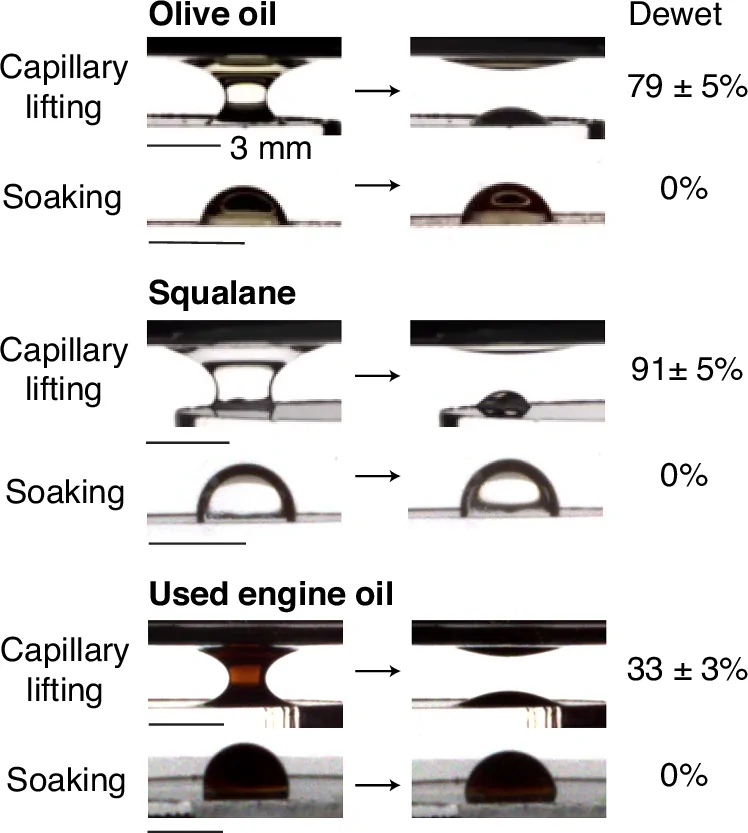
Comparison of cleaning effectiveness between capillary lifting and traditional soaking for real-world soil droplets. Experimental images showing the dewetting behaviour of olive oil, squalane, and used engine oil droplets during capillary lifting and soaking. Capillary lifting leads to substantial or partial droplet removal, whereas soaking alone does not induce dewetting. The percentages indicate the measured degree of dewetting for each system. All scale bars represent 3 mm. Error bars represent standard deviations.

Having demonstrated that our framework captures the dewetting behaviour in both pure liquids and formulations, we aim to move towards real-world cleaning situations through the capillary lifting mechanism. We do this by comparing the dewetting of common soils by capillary lifting and using the traditional soaking method with and without added detergent. For soils, we use olive oil (food residues), squalane (pharmaceutical, personal care and lubricants) and used engine oil (industrial soils with combustion residues). For the solid we use PMMA, a common, medium surface energy substrate, and pure water as the working liquid. For all soils, we can observe removal by capillary lifting, with dewetting efficiencies of 79 ± 5% for olive oil, 91 ± 5% for squalane, and 33 ± 3% for used engine oil. In contrast, when these soils are deposited on substrates in a water bath to simulate traditional soaking, they remain permanently adhered to the substrate (Fig. 5), despite some changes in the contact angles. The lower efficiency for used engine oil is likely due to the presence of solid residues, but still much better than for soaking. For consistency, we also performed soaking tests for the systems shown in Fig. 1 and Fig. 4, and similarly observed no detachment under static immersion (see details in Supplementary Note 11). Finally, taking olive oil as an example, adding a high concentration of surfactant to the soaking bath does lead to some removal, but only after ~1 hour, the time required for the surfactants to diffuse and interact with the droplet–fluid interface (as shown in Supplementary Note 12). This long timescale makes soaking much less efficient than capillary lifting.

## Conclusions

We have investigated capillary lifting mechanism for droplet dewetting in ternary system using a combination of experiments and computational modelling. Our results show that dewetting is favoured when ${\theta }_{{{\rm{dl}}}} > {\theta }_{{{\rm{t}}}}$. This corresponds to increasing *θ*_dl_ and hence decreasing *γ*_dg_/*γ*_lg_, offering an alternative to the use of surfactants to decrease the interfacial tension of the working liquid where air is available. For formulated fluids that often contain polymers and surfactants, we also show that molecular migration can significantly change the droplet dewetting outcome. Finally, we demonstrate the ability of capillary lifting to achieve significantly higher cleaning efficiency than soaking for real-world soils. This study advances the understanding of ternary dewetting mechanisms and highlights the potential of capillary lifting as an effective cleaning mechanism for situations with minimal energy and material input. Future studies will investigate the exploitation of related capillary-based phenomena to displace materials from solid surfaces, including solids, films, gels, and other viscoelastic materials. Our results show that the capillary lifting mechanism is most effective at low *Ca*, ideal for applications where a high level of control is needed or where resources are scarce. Future investigations will systematically explore the kinetic effects as the velocity of the rising working fluid is increased, including when formulated fluids containing polymers and surfactants are employed.

## Methods

### Experimental methods

#### Substrate and device preparation

Before each experiment, substrates and the dewetting device are cleaned sequentially with deionised water, isopropanol, and deionised water again, followed by drying with nitrogen gas. The fluid device features a central stage for substrate attachment, surrounded by four symmetric holes around it to ensure unform fluid distribution (Fig. 1). A 10 µL droplet is deposited on the substrate, and the working fluid is injected at a rate of 0.02 mL min^−1^ using a syringe pump (Pump 11 Pico Plus Elite, Havard Apparatus, Massachusetts, US), corresponding to a liquid rise velocity of 5.33 × 10^−7^ m s^−1^. The device base measures 25 × 25 mm and is capped with either a borosilicate (CM Scientific Ltd., Silsden, UK) or acrylic cubic square tube for visualization.

#### Imaging

The dewetting process was captured using: A colour digital camera (GV-79L0WP-C-HQ, IDS Imaging, Obersulm, Germany) with a 35 mm HP series lens (Edmund Optics Ltd., York, UK); A mono digital camera (UI-3880CP-M-GL Rev. 2, IDS Imaging, Obersulm, Germany) with a 6.5× zoom lens (MVL6X12Z, Thorlabs, New Jersey, US) and a 0.5× magnifying lens (MVL6X05L, Thorlabs, New Jersey, US). Lighting is provided by either an LED (RALENO, Amazon, UK) or a torch (ICEFIRE, Amazon, UK), coupled with a diffusive screen to ensure uniform illumination.

#### Materials

Substrates include stainless steel (SPI Supplies, West Chester, US), glass (LaboQuip, London, UK), PMMA (Sheet Plastics, Leicester, UK), PC (Sheet Plastics, Leicester, UK), and PTFE (Merck, Sigma-Aldrich, Gillingham, UK). Liquids used are deionised water (18.2 mW, Merck-Millipore, Hertfordshire, UK), tetradecane (Merck, Sigma-Aldrich, Gillingham, UK), decane (Merck, Sigma-Aldrich, Gillingham, UK), silicone oil (10 cSt, Merck, Sigma-Aldrich, Gillingham, UK), olive oil (Solesta, Aldi, Essen, Germany), squalane (Merck, Sigma-Aldrich, Gillingham, UK), and isopropanol (99.5% for HPLC, Thermo Scientific^TM^, Cambridge, UK). For visualisation in Fig. 1 of the main text, tetradecane droplets are dyed with Sudan IV (Merck, Sigma-Aldrich, Gillingham, UK) at a concentration of 1 mg in 6 mL, while water droplets are dyed with fluorescein sodium salt (Merck, Sigma-Aldrich, Gillingham, UK) at a concentration of 100 ppm. The formulation is composed of tripropylene Glycol Methyl Ether 0.45 wt%, diethylene glycol hexyl ether 0.45 wt%, ethylene glycol hexyl ether 0.45 wt%, and deionised water.

#### Interfacial measurements

Interfacial tension measurements were performed using the pendant drop method for binary systems and Neumann triangle analysis for ternary systems (see Fig. S7 in Supplementary Note 9). In the binary system, a tetradecane droplet is suspended from a needle immersed into the working fluid. The droplet shape is determined by the balance between interfacial tension and gravity, and used to extract the interfacial tension. The ternary system consists of the droplet, the working fluid, and the surrounding air. The Neumann triangle method is used to determine the relative interfacial tensions from the contact angles.

### Simulation methods

We employed Surface Evolver^21^ to model the dewetting behaviour of a droplet lifted from a solid substrate by an invading fluid under quasi-static conditions. In Surface Evolver, each surface or interface is discretised into triangular facets, and interfacial tensions are assigned based on experimental measurements obtained via techniques such as the pendant drop method or Neumann triangle analysis.

For the solid substrate and side walls, we ensured the chosen interfacial tensions obey the Young’s equation. For the droplet on the solid substrate, the relation is given by: *γ*_sd_ = *γ*_sl_ – *γ*_dl_ × cos*θ*_dl_, where *γ*_sd_, *γ*_sl_, *γ*_dl_ represent the interfacial tensions (or energies) for the solid–droplet, solid–liquid (working fluid), and droplet–liquid (working fluid) interfaces, respectively. *θ*_dl_ is the receding contact angle of the droplet on the substrate in the presence of the working fluid. For the working liquid on the side walls, the contact angle is set to *θ*_lw_ = 90.0°, such that *γ*_lw_ = *γ*_wg_ – *γ*_lg_ × cos*θ*_lw_ leads to *γ*_lw_ = *γ*_wg_, where *γ*_lw_ and *γ*_wg_ refer to the interfacial tensions (or energies) for the liquid–wall and wall–gas interfaces, respectively.

In the model, measured interfacial tensions *γ*_dg_ and *γ*_dl_ are normalised by *γ*_lg_ before being input into the simulation, with *γ*_sl_/*γ*_lg_ and *γ*_wg_/*γ*_lg_ set to 1. Densities are normalised by the density of the working liquid. To match the experimental conditions, the gravitational strength *g* is set by matching the Bond number (Bo) of the experiment. The droplet volume remains constant, consistent with experimental conditions. The initial volume of the invading fluid is set to establish a stable liquid bridge between the air and the substrate. The fluid is then added incrementally, followed by energy minimization at each step. The equilibrium state from each step serves as the initial condition for the next, simulating the continuous invasion process under quasi-static conditions.

## Supplementary information

Transparent Peer Review file

Supplemental material for publication

## Data Availability

The data that support the findings of this study are available in Durham University research data repository at https://doi.org/10.15128/r3f1881k890.

## Code availability

Simulations are performed using Surface Evolver version 2.70. A representative Surface Evolver script used in this study is available in the Durham University research data repository at https://doi.org/10.15128/r3f1881k890.
