# Supplementary material for: Droplet removal by capillary lifting
Source: Commun Phys. 2026 Jun 12;9(1):307. doi: 10.1038/s42005-026-02714-4 (PMC13616733; doi:10.1038/s42005-026-02714-4)
Supplement: Supplementary file 1 [file 42005_2026_2714_MOESM1_ESM.pdf]

nature portfolio

Peer Review File

Droplet Removal by Capillary Lifting

Corresponding Author: Professor Kislon Voitchovsky

This file contains all reviewer reports in order by version, followed by all author rebuttals in order by version.

Attachments originally included by the reviewers as part of their assessment can be found at the end of this file.

Version 0:

Reviewer comments:

Reviewer #1

(Remarks to the Author)
In the present manuscript, "Droplet Removal by Capillary Lifting" the authors study capillary lifting mechanism for droplet dewetting in ternary system. Using a unique experimental system, the authors demonstrate either dewetting or non-dewetting of soil droplets on a substrate using a suitable working liquid. They find that dewetting is favored when the working liquid has a high interfacial tension, enabling the capillary force to effectively lift the droplet. The idea proposed by the authors is novel and good, which will be of interest to the wider readership of the journal. However, I feel that at places the paper is written rather poorly and lacks clarity which will be very difficult for readers to understand. In addition, there a few major scientific/technical points, as listed below, that should also be addressed before I recommend it for publication in Communications Physics.
1. The authors have used a very unconventional experimental system to study, hence more detailed and clear experimental procedure and defining experimental parameters needs to be provided. The current description in the Methods section and the drawing image shown in Fig. S1 are not sufficient for readers to appreciate the experimental procedure.
2. Description of various interfacial tensions in Fig 1(a) is very poorly defined. It is very confusing to understand why there would be a droplet-gas interface (yellow color region in Fig 1 (a) & (b simulation)) when the droplet is surrounded by the working liquid. I don't see a similar droplet-gas interface in the corresponding experimental images in Fig 1(b). Again, this is due to the poor description of the experimental system as mentioned in the last point. This part needs to be thoroughly revised.
3. The authors mentioned that they studied dewetting in ternary systems, which involves three fluid components and a solid. Is this is a three-fluid system with a substrate or just two fluid phases and air/gas? For later case, the author should define the system as three-phase system rather than three fluid system, which is misleading.
4. Any reason why none of the simulations could capture partial dewetting. All simulation results show either complete dewetting or no dewetting, while experiments also show partial dewetting. Is it due to the finite surface roughness/heterogeneity which is not included in simulations?
5. What is being varied in Fig 1 (b) & (c) from first to the third frame? Is it only the time, or the height of working fluid, or both? This needs to be mentioned in the figure.
6. The authors have used variety of substrates, PTFE, stainless steel, PC, PMMA and glass to demonstrate dewetting led capillary removal of soil drops. However, it is well known that any application of contact line motion, including dewetting, depends strongly on the surface roughness of the underlying solid substrate. Still the authors never quantified surface roughness. In addition to the static contact angles (as shown in Fig. S2 and S4), the authors should also report the contact angle hysteresis of all the soil liquids on different substrates.
7. On one hand, the authors report the contact angle of tetradecane on PMMA as approximately 0° in Fig S2 (a), but in Fig. S2 (b) the contact angle of water on PMMA is reported as 71.22 ± 3.88° . The author should maintain uniformity (in terms of significant digits) in reporting experimental values. Is it true that the authors can resolve optical contact angle with second decimal place resolution, hard to believe.
8. On Page 2 last line, the authors write, "The simulated evolutions of the interface configuration agree well with experiments, as shown in Fig. 1(b-c)." It is known that the Surface Evolver provides equilibrium configuration as a result of energy minimization. So how can the authors investigate the "evolutions of the interface configuration", as it is a dynamic process.
9. On Page 5, second paragraph, the authors write, "When a droplet forms a capillary bridge between the solid and the air interface,…..". How can a droplet form capillary bridge between a solid and air interface, as at the air side the droplet will form curved interface due to surface tension. To form a capillary bridge, a droplet should have two interfaces on both side.

Again it is not clear due to poor experimental description.

Reviewer #2

(Remarks to the Author)
Report on the manuscript "Droplet Removal by Capillary Lifting" by Kae Sun et al.

This manuscript studies the removal of liquid droplets from surfaces using an immiscible working liquid.

I read the article twice and did not fully understand it. The figures are unclear (e.g., the formation of the gas layer in Figure 1). Much information is missing. The key parameter governing the physical process of droplet removal is the force of gravity. The article does not specify the density of the liquid. How can readers understand this article? In my opinion, the roughness of the solid surface should be indicated, as it can affect the attachment of the droplet contact line to the surface.

I do not recommend this article for publication in the present form.

Reviewer #3

(Remarks to the Author)
In this work, droplet removal from solid surfaces was studied by immersing the droplets in a working fluid without surfactants. A fluidic device was employed to quasi-statically raise the working liquid, thereby creating a liquid bridge (surrounded by the working fluid) between the air interface and the solid surface, which facilitated the removal of sessile droplets. Experiments were conducted on a range of substrates, with variations in both the working liquids and the fluids forming the droplets. The process was also analyzed theoretically using Surface Evolver under quasi-static conditions, accounting for interfacial energies and gravity. A wetting-dewetting diagram was obtained based on the two characteristic contact angles of the liquid bridge confined between the two heterogeneous interfaces: $\cos\theta_{dl}$ (liquid-droplet-solid) and $\cos\theta_t$ (liquid-droplet-air). Dewetting was found to be favored when $\cos\theta_{dl} < \cos\theta_t$. The dewetting method of capillary lifting was subsequently compared with detergent soaking experiments, and was found to provide higher removal efficiency. Although this study systematically demonstrates the mechanism of droplet removal by capillary lifting through experiments supported by Surface Evolver simulations, the wetting–dewetting phenomena may also be interpreted through a thermodynamic analysis of spreading coefficients ($\cos\theta_{dl} \sim -1$). Furthermore, if the raising rate of the working fluid is sufficiently high, a transient liquid bridge may not form, and the dewetting process instead corresponds to the contact angle increasing to 180°. The authors should examine whether the raising rate influences the wetting-dewetting diagram. If it does not, the capillary lifting method can be regarded as thermodynamically driven, and the liquid bridge plays no significant role; otherwise, it is kinetically driven, and the formation of the liquid bridge becomes important. Since the overall contribution of the work is not yet clear, I cannot recommend this manuscript for publication in its current form (rejection is suggested, with resubmission recommended after major revision). Nevertheless, the manuscript would benefit from addressing the following points to improve clarity and strengthen its presentation.

1. The manuscript focuses on the contact angle of the droplet in liquid (θ_{dl}) and the effective top contact angle (θ_t) in establishing the dewetting criterion. However, during the actual removal process, the sessile droplet contact angle is expected to increase gradually as the working liquid rises. Reporting such dynamic contact angle data would strengthen the interpretation of the dewetting mechanism.
2. For the wetting scenario (in contrast to the dewetting case), the time evolution of the dynamic contact angle should be reported as the working liquid rises. Presumably, the contact angle will eventually reach a constant value.
3. The manuscript does not present a systematic study of capillary lifting using aqueous surfactant solutions at different concentrations, but instead relies on a low-concentration glycol ether formulation to tune interfacial tensions. Employing the same working fluids in both soaking and capillary lifting experiments would allow for a more meaningful comparison between the two approaches.
4. The study employs a quasi-static lifting process, but no comparison is provided for different lifting speeds (faster vs. slower rise). Such tests would help distinguish whether the observed dewetting is governed primarily by quasi-static thermodynamic equilibrium or whether kinetic effects significantly influence the outcome.
5. I suggest that the authors clarify whether a simple bulk immersion test, in which the droplet is directly deposited through the working liquid bath onto the solid surface, yields the same equilibrium configuration as observed in the wetting or dewetting scenario. Such a check would help determine whether the mechanism is fundamentally thermodynamic in nature or dependent on the kinetic lifting process.
6. The manuscript relies primarily on 10 μL droplets, where the capillary length is comparable to the droplet size. As shown in Figure S3, increasing droplet size leads to higher dewetting percentages due to gravitational effects. Since droplet size affects the balance between capillary and gravitational forces, it would be important to discuss whether the applicability of dewetting phenomena extends across a broader range of droplet volumes.
7. In the Surface Evolver simulations, is the solid-liquid interfacial tension (or $\cos\theta$) obtained directly from the same experiments rather than determined independently? If so, does this approach yield the correct solid-liquid tension for the dewetting case?
8. The formation of the unstable liquid bridge is indeed interesting; however, it is inherently coupled with the rising working fluid due to the presence of another air–liquid interface. This process may provide a pathway to overcome the kinetic barrier, which should be explained in greater detail. In addition, regime V of Figure 2f cannot be explained solely by free-energy minimization and should likewise be discussed in more detail.

Version 1:

Reviewer comments:

Reviewer #1

(Remarks to the Author)
The authors have carefully and comprehensively addressed all the concerns raised during the review process. Their responses are clear, well-reasoned, and adequately supported by appropriate revisions to the manuscript. The modifications have significantly improved the clarity and overall quality of the work. In its current form, the manuscript meets the standards for publication, and I am satisfied with the revisions. I therefore recommend that it be accepted for publication.

Reviewer #2

(Remarks to the Author)
All my comments have been addressed, and I appreciate the explanations given by the authors in their response to my comments.

Reviewer #3

(Remarks to the Author)
The authors have adequately addressed my previous query by claiming that the capillary lifting mechanism is thermodynamically driven. However, this is achieved by deliberately restricting the experimental setup to a strictly quasi-static limit with an ultra-slow fluid raising velocity of about 5×10-7 m/s. Furthermore, as the revised manuscript indicates, the dewetting efficiency decreases significantly when the velocity increases and the system enters a kinetic regime. The sharp decline in efficiency under dynamic conditions represents a fundamental flaw in the manuscript’s core premise.
Since a true thermodynamic transition should be path-independent (and thus independent of the lifting velocity), the observed rate-dependency suggests that the underlying mechanism is kinetically limited rather than purely thermodynamic. This indicates that the proposed "capillary lifting" is not a robust removal mechanism, but a metastable state that is easily disrupted. As velocity increases, hydrodynamic effects likely force the capillary bridge to rupture prematurely, preventing complete dewetting. Furthermore, the requirement for an exceptionally slow and precisely controlled displacement rate to maintain these quasi-static conditions poses significant practical
challenges. Given this lack of robustness under dynamic conditions and the resulting limited technological relevance, I cannot recommend the manuscript for publication in its current form.

Version 2:

Reviewer comments:

Reviewer #3

(Remarks to the Author)
The authors’ responses to my comments are now acceptable, and the manuscript can be published.

Open Access This Peer Review File is licensed under a Creative Commons Attribution 4.0 International License, which permits use, sharing, adaptation, distribution and reproduction in any medium or format, as long as you give appropriate credit to the original author(s) and the source, provide a link to the Creative Commons license, and indicate if changes were made.
In cases where reviewers are anonymous, credit should be given to 'Anonymous Referee' and the source.
The images or other third party material in this Peer Review File are included in the article's Creative Commons license, unless indicated otherwise in a credit line to the material. If material is not included in the article's Creative Commons license and your intended use is not permitted by statutory regulation or exceeds the permitted use, you will need to obtain permission directly from the copyright holder.
To view a copy of this license, visit https://creativecommons.org/licenses/by/4.0/

We would like to thank the reviewers for their helpful comments and suggestions. We have addressed these comments in full, as detailed hereafter.

We also would like to draw their attention to the fact that the affiliation of one of the authors has been updated to reflect a new position.

For the sake of clarity, the Referees' comments are shown in black, our response appear in blue and any resulting change in the manuscript and supplementary material is shown in red.

Reviewers' comments:

Reviewer #1 (Remarks to the Author):

In the present manuscript, "Droplet Removal by Capillary Lifting" the authors study capillary lifting mechanism for droplet dewetting in ternary system. Using a unique experimental system, the authors demonstrate either dewetting or non-dewetting of soil droplets on a substrate using a suitable working liquid. They find that dewetting is favored when the working liquid has a high interfacial tension, enabling the capillary force to effectively lift the droplet. The idea proposed by the authors is novel and good, which will be of interest to the wider readership of the journal. However, I feel that at places the paper is written rather poorly and lacks clarity which will be very difficult for readers to understand. In addition, there a few major scientific/technical points, as listed below, that should also be addressed before I recommend it for publication in Communications Physics.

We thank the reviewer for these encouraging comments. We have now extensively revised the manuscript, including the rewriting of certain parts that were previously confusing.

1. The authors have used a very unconventional experimental system to study, hence more detailed and clear experimental procedure and defining experimental parameters needs to be provided. The current description in the Methods section and the drawing image shown in Fig. S1 are not sufficient for readers to appreciate the experimental procedure.

We thank the reviewer for point out this gap in our presentation. We agree that the experimental design and relevant parameters needed clarification. We have made the following changes:

(1) We have rewritten the lead-in paragraph in the Results section to more clearly explain the experimental design and highlight the key steps and parameters involved: "To investigate capillary lifting in ternary systems, we employed an integrated experimental–simulation approach. Details of the experimental procedure can be found in Methods. In brief, we designed a fluidic device (Fig. 1a) in which a droplet (~10 µL) is deposited on a central substrate. The working fluid is injected from below through four symmetrically arranged holes using a syringe pump, ensuring uniform lateral distribution and a controlled rise velocity of ~5 × 10^{-7} m/s, and allowing the system to operate near quasi-equilibrium (see details of kinetic investigation in Fig. 2 and associated text). As the working fluid rises, the droplet forms a capillary bridge connecting the bottom substrate and the top air–

droplet interface, while being laterally surrounded by the working fluid. This resembles classical capillary bridges between parallel solid plates, except that here the air interface acts as a deformable soft surface; we therefore refer to this structure as a capillary bridge. The bridge geometry evolves during fluid injection and is recorded using a dual-camera system. The main camera captures the bridge shape, allowing direct comparison with simulation profiles, while the side camera provides a complementary perspective from a slightly elevated angle, clearly revealing the droplet–air interface (as shown in Fig. 1a and detailed in Fig. S1 of Supplementary Material (supplementary section SM) 1). This experimental setup enables us to capture the full evolution of the bridge, including its deformation and potential rupture, which ultimately leads to distinct dewetting outcomes."

(2) Figure 1 has been updated with clearer annotations and a more detailed schematic of the experimental configuration. The caption has also been revised to provide a concise description of the setup and procedure. "

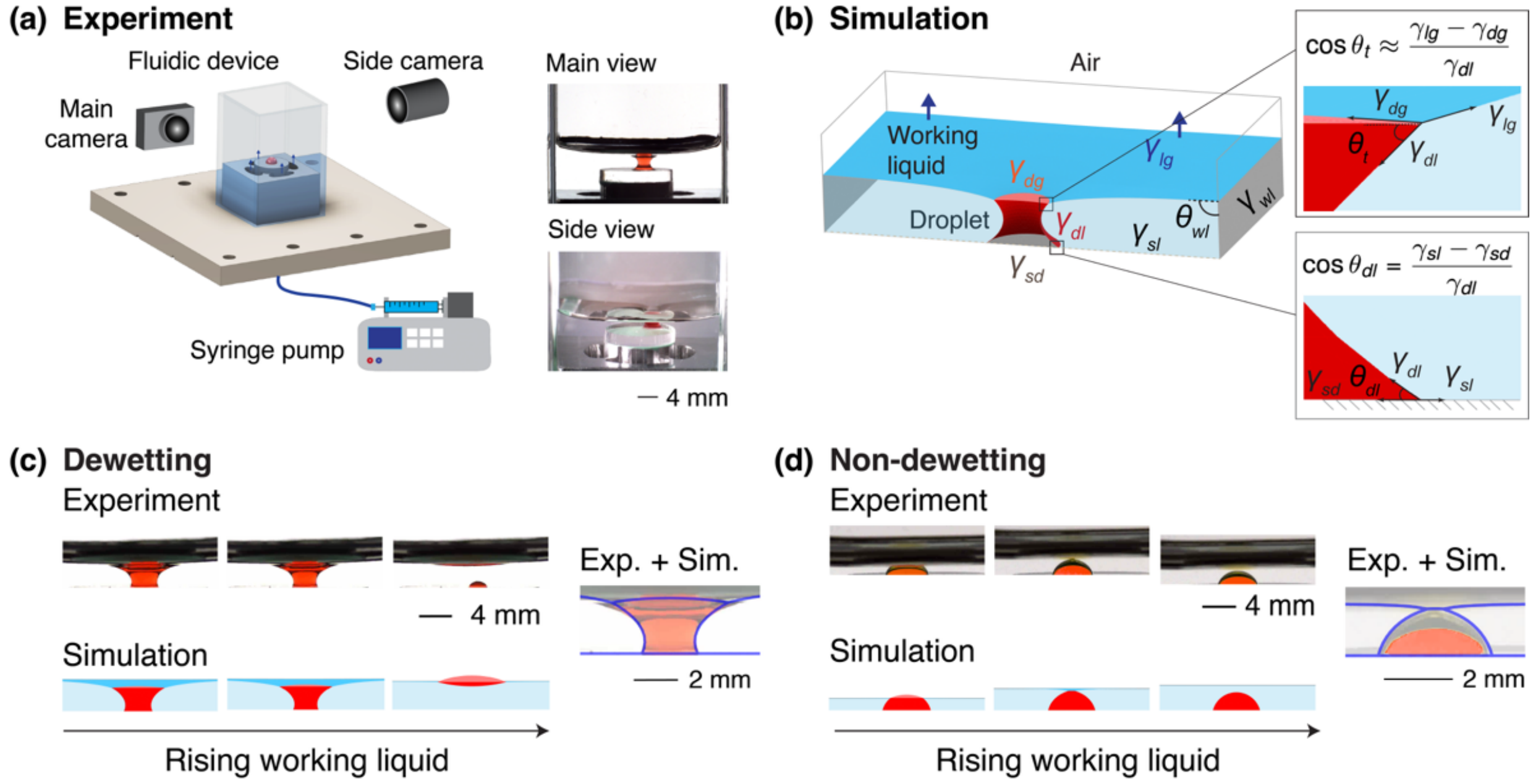

Figure 1. Capillary lifting of a droplet from a surface by a rising working liquid. (a) Experimental setup consisting of a dewetting device, a syringe pump, and a dual-camera imaging system. In the dewetting device, the central pillar supports the soil droplet, with the working fluid is injected from below by a syringe pump. The main camera records the droplet profile as its geometry evolves during removal, whereas the auxiliary side camera is positioned slightly above the substrate plane to visualise the droplet–air (and liquid–air) interface. **(b)** Simulation schematic diagram of a soil droplet being lifted from a solid substrate by a rising working liquid. The relevant thermodynamics parameters are the interfacial tensions (or energies) of the solid substrate–droplet γ_{sd} (grey), droplet–gas γ_{dg} (light red), liquid–gas γ_{lg} (blue), substrate–liquid γ_{sl} (hidden beneath liquid), droplet–liquid γ_{dl} (red), liquid–wall γ_{lw} (grey), and wall–gas γ_{wg} (transparent). The receding contact angle of the droplet in liquid is θ_{dl} and the effective top contact angle is θ_t, which can be expressed in terms of the balance of interfacial tensions (see insets). **(c–d)** Examples of capillary lifting observed experimentally and in simulations for two typical situations. For the combined simulation and experimental panels, the simulated profiles are shown as blue lines atop the experimental results. (c) Successful dewetting of a dyed tetradecane droplet on a polymethyl methacrylate (PMMA) surface

by water. (d) Failed dewetting of a dyed water droplet on PMMA by tetradecane. The dyed water droplet is colourised red for visual consistency (naturally yellow)."

(3) We now also include a new supplementary material section (supplementary section SM 1) containing complementary figures that illustrate the time evolution of droplets during the capillary lifting process. These images provide visual clarity and are intended to help readers better understand the experimental observations. "

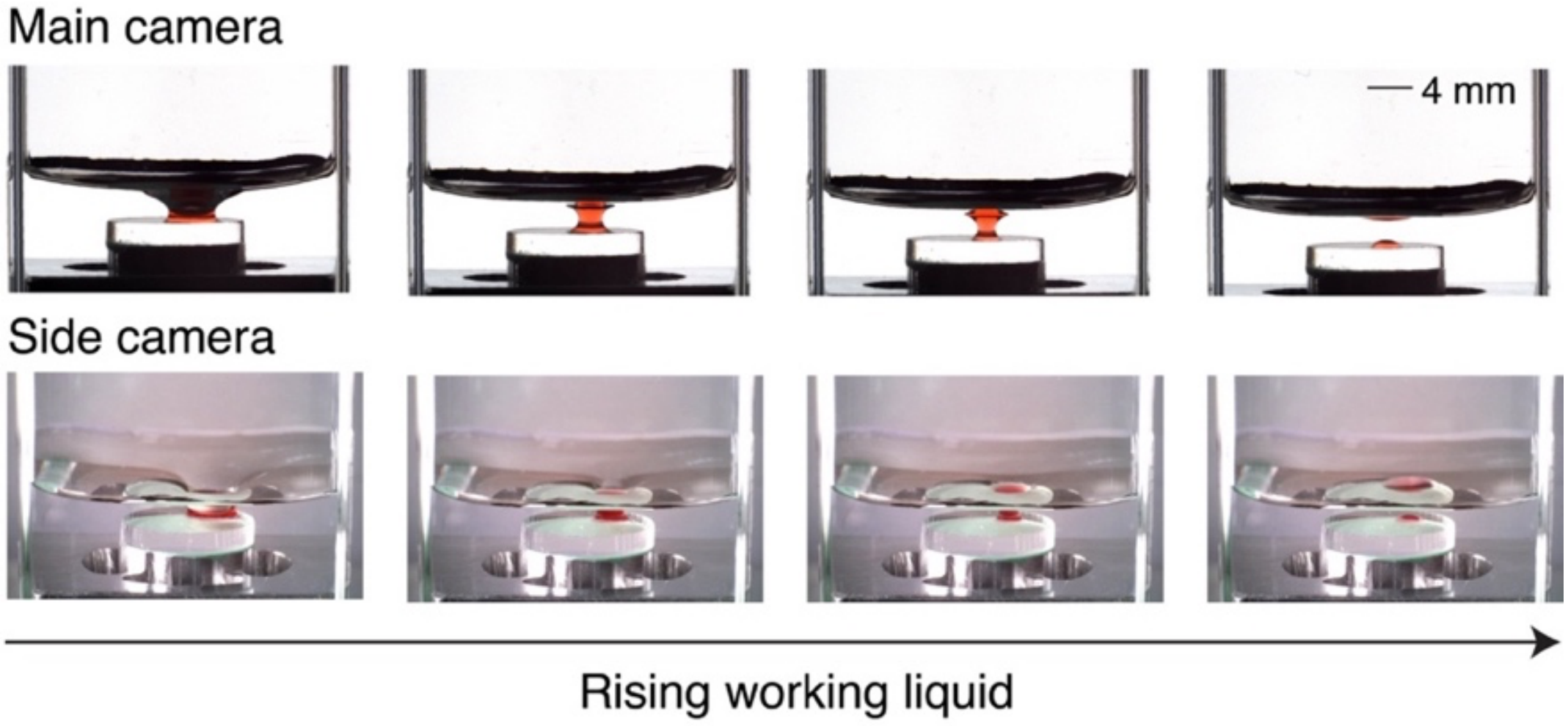

Fig. S1. The imaging system used to observe capillary lifting. The main camera captures the full droplet configuration during lifting (top panel), while a slightly elevated side camera provides a complementary view from another angle to detect any asymmetric behaviour due to pinning (bottom panel). This side view also clearly reveals the droplet–air interface, corresponding to the top of the defined capillary bridge between air and substrate. The shown field of view of the main camera has been readjusted in each image for better visibility"

2. Description of various interfacial tensions in Fig 1(a) is very poorly defined. It is very confusing to understand why there would be a droplet-gas interface (yellow color region in Fig 1 (a) & (b simulation)) when the droplet is surrounded by the working liquid. I don't see a similar droplet-gas interface in the corresponding experimental images in Fig 1(b). Again, this is due to the poor description of the experimental system as mentioned in the last point. This part needs to be thoroughly revised.

The capillary bridge in our experiments is laterally surrounded by the working fluid, while its top is directly exposed to air, resulting in a droplet–gas interface. In the previous version of Fig. 1a, this top droplet–gas interface was highlighted in yellow for clarity, whereas experimentally it appears as the same color as the droplet (e.g. red in the dewetting case). This interface is not directly visible in the main experimental images for two reasons: First, the main camera captures the front profile of the capillary bridge, not the top; second, the curvature of the working fluid at the wall (shown as black in the experimental images Fig. 1c-d) obscures the top droplet–gas interface.

The revisions made in response to Question 1 also addressed the confusion correctly raised by the reviewer in Question 2. Specifically, the three following summarised below to avoid repetition:

(1) Figure 1and caption: These have been revised to clarify the experimental setup and to distinguish the various interfaces. A secondary, slightly angled camera view is now included to capture the top of the droplet, allowing clear visualisation of the droplet–gas interface.

(2) Consistent colouring: In all images in Fig. 1, the droplet–working fluid interface is now shown in red, and the droplet–gas interface in light red. This aligns the visual representation with experimental observation and avoids any potential misinterpretation caused by colour schemes.

(3) New Supplementary material section SM 1: A new SI figure fully documents the evolution of the droplet during capillary lift-off using the dual-camera system. This allows unambiguous distinction of the top droplet–gas interface both during bridge formation and in the final dewetting state, where the detached droplet clearly exhibits its droplet–gas interface in red.

3. The authors mentioned that they studied dewetting in ternary systems, which involves three fluid components and a solid. Is this a three-fluid system with a substrate or just two fluid phases and air/gas? For later case, the author should define the system as three-phase system rather than three fluid system, which is misleading.

We understand that the terminology may have caused some confusion. The 'ternary' system we refer to consists of the droplet, the working liquid, and the gas phase. We used the term 'fluid' because it includes both gases and liquids. We are concerned that using the term 'phase' would complicate the explanation because the system comprises four phases: two liquids, one gas and one solid.

To improve clarity, in the first mention of ternary systems (following the description of binary systems), we revised the statement: "In contrast, significant gaps remain in our understanding of dewetting in ternary systems where three fluid components are present together with the solid." Furthermore, in the next paragraph, when we first introduce the specific ternary system in this work, we explicitly list the three fluids in parentheses to avoid ambiguity: "In this study, we explore a fundamental mechanism of ternary dewetting (involving the droplet, the working liquid, and the gas phase) that we term capillary lifting…". These revisions ensure that "ternary" is clearly defined and consistent throughout the manuscript.

4. Any reason why none of the simulations could capture partial dewetting. All simulation results show either complete dewetting or no dewetting, while experiments also show partial dewetting. Is it due to the finite surface roughness/heterogeneity which is not included in simulations?

Our simulations do capture partial dewetting. This is discussed in the Results section where we describe the transition region between dewetting and non-dewetting: "We now focus on the

sharpness of the boundary between full and non dewetting as predicted by the computational model. An example of a partial dewetting simulation is provided in Fig. S6 (see section supplementary 7). When all interfacial tensions are held fixed (arrow in Fig. 3b), the transition typically follows a smooth hyperbolic tangent profile when varying θ_{dl} (Fig. 3f). In contrast, when θ_{dl} is held constant and the interfacial tension ratio γ_{dg}/γ_{lg} varies (arrow in Fig. 3c), the transition becomes more abrupt and sharpens further with increasing θ_{dl} (Fig. 3g). These findings suggest that efficient dewetting can be achieved at relatively low contact angles by tuning interfacial tensions in a ternary system. For instance, abrupt dewetting transitions can be triggered by a slight decrease of γ_{dg}/γ_{lg}." The intermediate data points in Fig. 3f and 3g correspond to partial dewetting cases.

Partial dewetting via simulation is also included in Supplementary Material section SM 7, where the transition curve in Fig. S5 includes yellow and light blue datapoints representing partial dewetting. We have now added a new simulation snapshot (Fig. S6) showing explicitly the partial dewetting in supplementary Material, section SM 7: "Here we show example partial dewetting simulation snapshots illustrating the configuration evolution of the capillary bridge as it stretches and ruptures, ultimately leading to the separation of the droplet into two individual bodies.

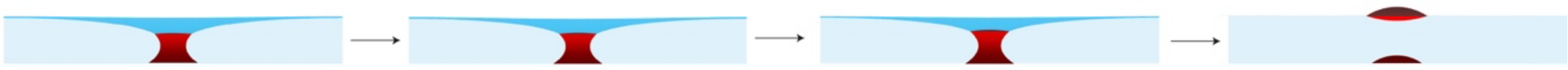

Fig. S6. Example of simulation results showing partial dewetting. The system parameters are γ_{dg}/γ_{lg}=0.4; γ_{dl}/γ_{lg}=0.7; θ_{dl}=35.0°. From left to right, the working liquid rises."

In the simulations partial dewetting occurs within a narrow range of parameters. We believe this is because the substrate is idealised as perfectly smooth and homogeneous. In contrast, surface roughness and chemical heterogeneity are ubiquitous in the experiments. We agree with the reviewer that this makes partial dewetting more prominent in the experiments: "…We note that the partial dewetting states captured in the simulation model (Figs. 3f–g and supplementary section SM 7) occur only within a very narrow region of interfacial parameter space."

Also: "…One important assumption of the model is that of perfectly smooth and homogeneous surfaces. However, experimentally most surfaces inevitably exhibit some roughness and chemical heterogeneities. As a result, we typically observe contact line pinning which leads to residues remaining on the surface upon dewetting and a slight deviation in the percentage of removal between experiments and simulations (as shown in Fig. 1c and the experimental–numerical comparison in supplementary section SM 8). Consequently, partial dewetting is more prominent in experiments than in simulations."

5. What is being varied in Fig 1 (b) & (c) from first to the third frame? Is it only the time, or the height of working fluid, or both? This needs to be mentioned in the figure.

The variation across the frames in Fig. 1b and Fig. 1c corresponds to both time and the increasing height of the working liquid, which is injected into the fluidic device. We have now added an arrow indicating "Rising working liquid" in Fig. 1c and Fig. 1d to make this explicitly clear.

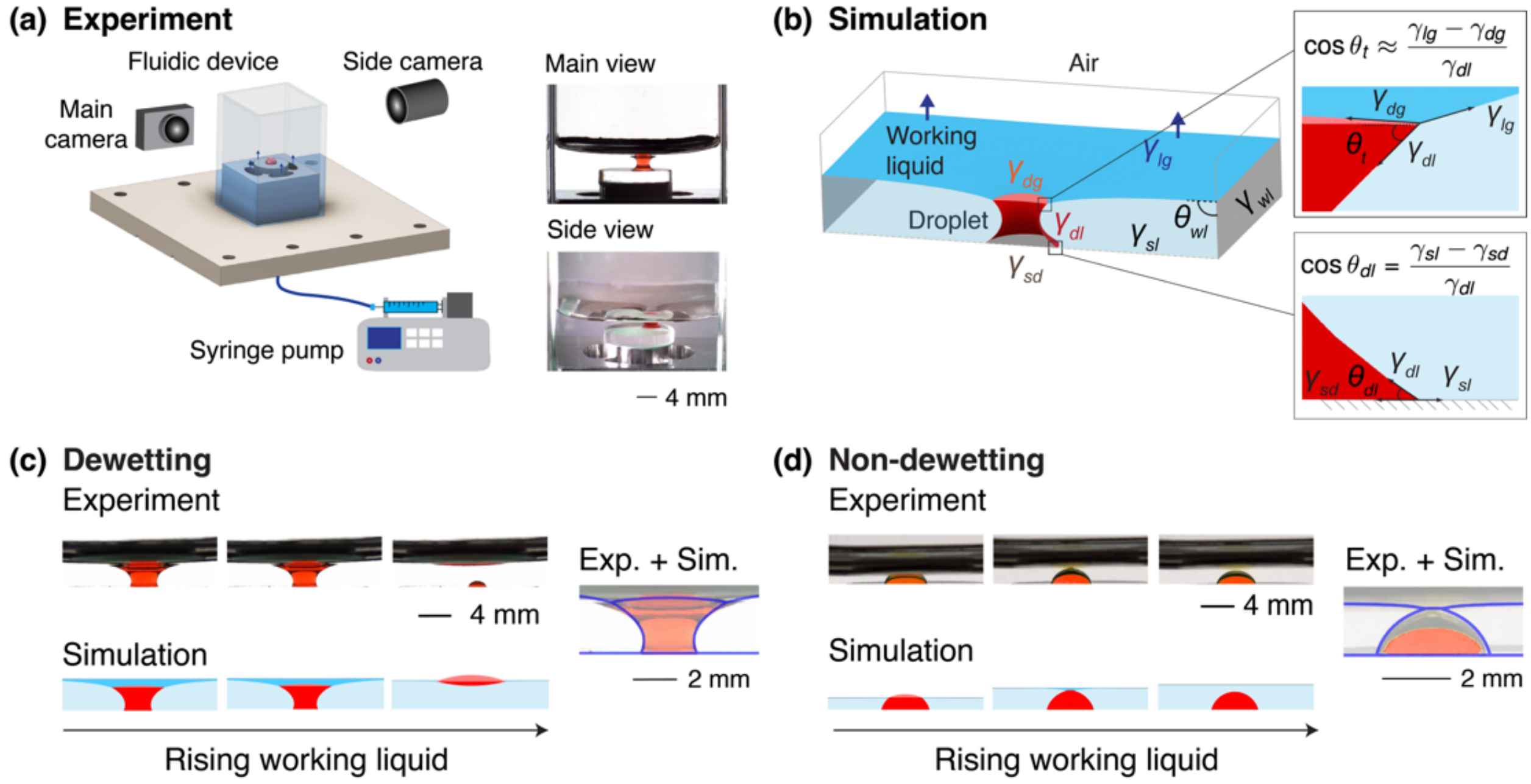

6. The authors have used variety of substrates, PTFE, stainless steel, PC, PMMA and glass to demonstrate dewetting led capillary removal of soil drops. However, it is well known that any application of contact line motion, including dewetting, depends strongly on the surface roughness of the underlying solid substrate. Still the authors never quantified surface roughness. In addition to the static contact angles (as shown in Fig. S2 and S4), the authors should also report the contact angle hysteresis of all the soil liquids on different substrates.

We thank the reviewer for this helpful suggestion. We have now added measurements of both the surface roughness and the contact angle hysteresis for the different substrates. They appear in a new Supplementary Material, section SM 6 “Substrates roughness and contact angle hysteresis”, and summarised in tables III and IV:

“As described in the main text, we used commercially available substrates to validate the effect of capillary lifting in practical cleaning. These surfaces are not ideal, exhibiting both topographic and chemical heterogeneities. Here, we characterised the substrates using contact angle hysteresis (CAH) measurements and atomic force microscopy (AFM) imaging.

In the CAH measurements, droplets were deposited on the substrates while immersed in the working liquid, and the droplet was injected and withdrawn obtain advancing and receding angles (Table III). Measurements under liquid are inherently much more challenging than measurements in air due to droplet mobility upon needle contact, non-uniform pinning along the contact line, gravitational deformation, and substrate heterogeneity across different measurement locations. These factors contribute to the relatively large uncertainties. In Table II, the advancing and receding angles were determined directly from video frames at the moments when the contact line was advancing or receding, with standard deviations calculated from three repeated measurements.

For the capillary bridge simulations, we used the receding contact angle observed from the corresponding capillary bridge retraction experiment. The values lie within the range of the experimentally measured CAH for the droplet setup (as reported in Table II). This means the

measurements remain sufficiently reliable for estimating the relevant contact angles, which is further supported by the good agreement between the experimental and simulated capillary bridge morphologies.

TABLE III. Advancing and receding contact angle measurements in the surrounding liquid. All measurements were repeated at least three times to determine the standard deviation.

Surface	Droplet composition	Surrounding liquid	Advancing angle ± standard dev. (°)	Receding angle ± standard dev. (°)
Stainless Steel	Tetradecane	Water	118.2 ± 6.5	37.0 ± 7.1
Stainless Steel	Decane	Water	125.1 ± 6.0	53.4 ± 5.8
Stainless Steel	Tetradecane	Formulation	118.7 ± 3.9	30.8 ± 1.4
Glass	Decane	Water	132.6 ± 5.6	65.8 ± 1.3
Glass	Tetradecane	Water	132.2 ± 5.5	57.9 ± 1.4
Glass	Silicone oil	Water	129.2 ± 6.5	118.7 ± 4.9
Glass	Water (fluorescein dyed)	Tetradecane	81.2 ± 8.2	75.5 ± 11.3
PTFE	Tetradecane	Water	28.2 ± 3.4	13.0 ± 2.0
PTFE	Tetradecane	Formulation	32.0 ± 9.8	25.0 ± 4.8
PTFE	Decane	Water	26.0 ± 3.6	15.2 ± 2.0
PTFE	Decane	Formulation	65.1 ± 4.4	40.4 ± 2.8
PMMA	Water (fluorescein dyed)	Tetradecane	98.6 ± 8.9	88.5 ± 3.1
PMMA	Tetradecane	Formulation	106 ± 20.3	130.0 ± 4.6
PMMA	Tetradecane	Water	126.5 ± 7.0	55.6 ± 8.1
PMMA	Squalane	Water	119.6 ± 3.8	59.6 ± 6.0
PMMA	Olive oil	Water	113.0 ± 16.8	50.5 ± 6.0
PMMA	Decane	Water	89.1 ± 6.1	59.6 ± 4.4
PMMA	Tetradecane (Sudan IV dyed)	Water	81.1 ± 11.1	71.7 ± 12.2
PC	Tetradecane	Formulation	113.6 ± 9.4	50.2 ± 4.3
PC	Tetradecane	Water	73.9 ± 8.9	19.9 ± 3.7
PC	Decane	Water	78.0 ± 2.0	22.2 ± 5.1

AFM imaging was performed in contact mode using a JPK NanoWizard 3 (Bruker, USA) at ambient temperature and humidity. The AFM tip/cantilever (SNL-10 tip A, Bruker AFM Probes, USA, nominal spring constant 0.4 N/m) was calibrated using its thermal spectrum after the measurements. Representative AFM images and the corresponding roughness parameters (average ± standard deviation) were obtained from three repeated measurements across randomly selected areas of each sample. Roughness statistics were processed using Gwyddion[5] (Table IV).

TABLE IV. AFM topographic images of the substrates and corresponding roughness parameters. Each measurement was repeated at least three times to determine the standard deviation.

Material	AFM Topographic image	Mean roughness (Sa) in nm	RMS roughness (Sq) in nm	Surface area/projected area (r)
Stainless steel	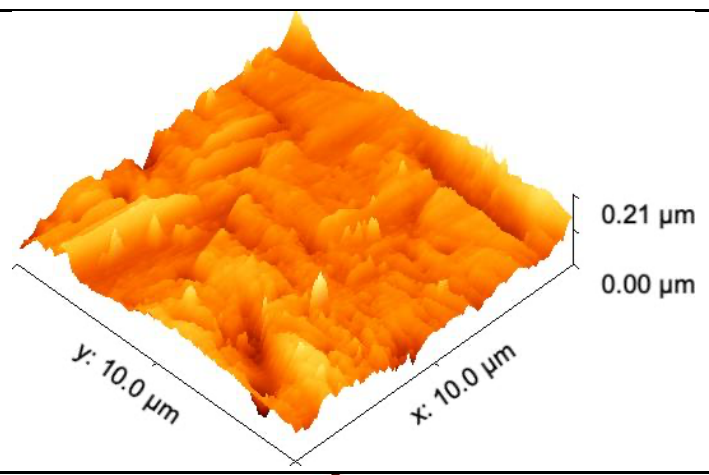	24.13 ± 13.38	33.37 ± 21.10	1.032 ± 0.030
Glass	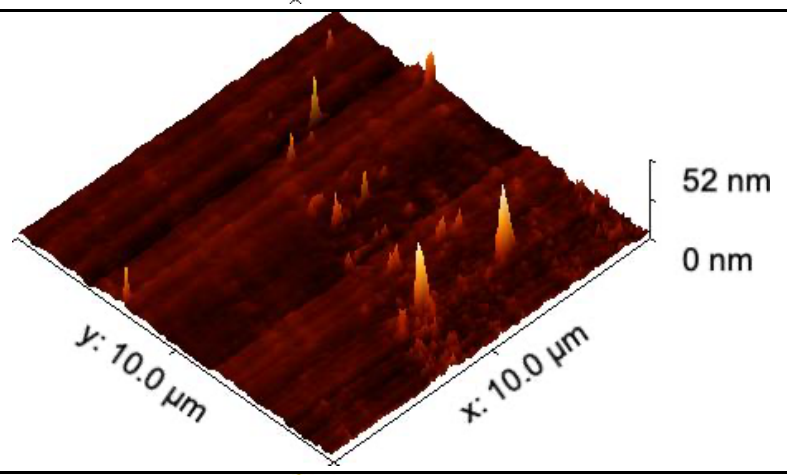	1.26 ± 0.39	1.71 ± 0.65	1.0004 ± 0.001
PMMA	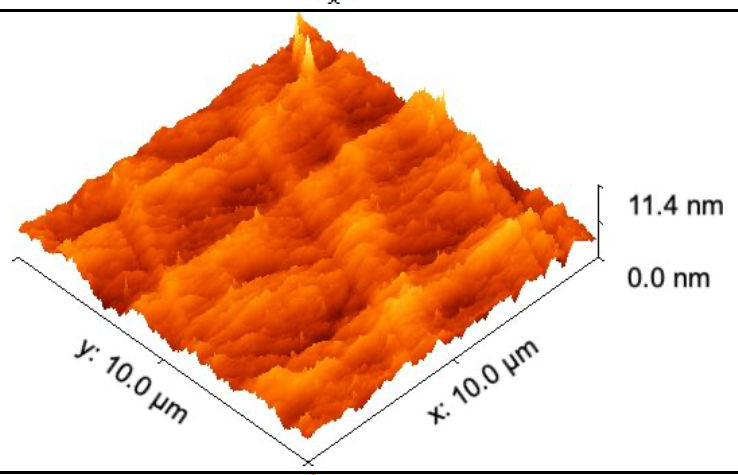	0.97 ± 0.18	1.62 ± 0.60	1.000 ± 0.001
PC	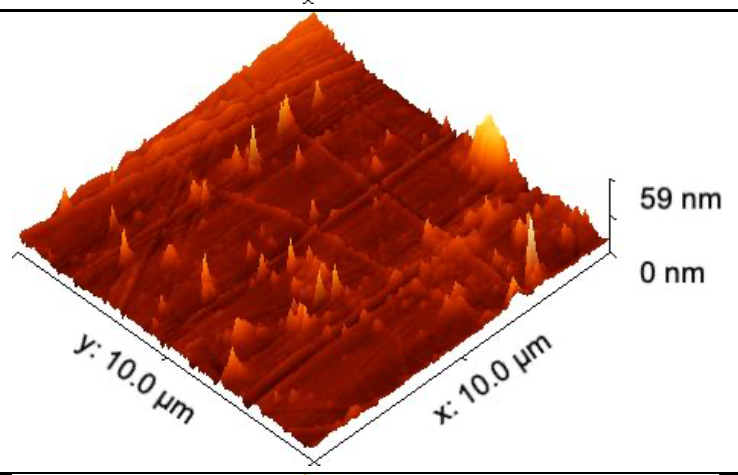	2.44 ± 0.58	4.21 ± 1.29	1.003 ± 0.002
PTFE	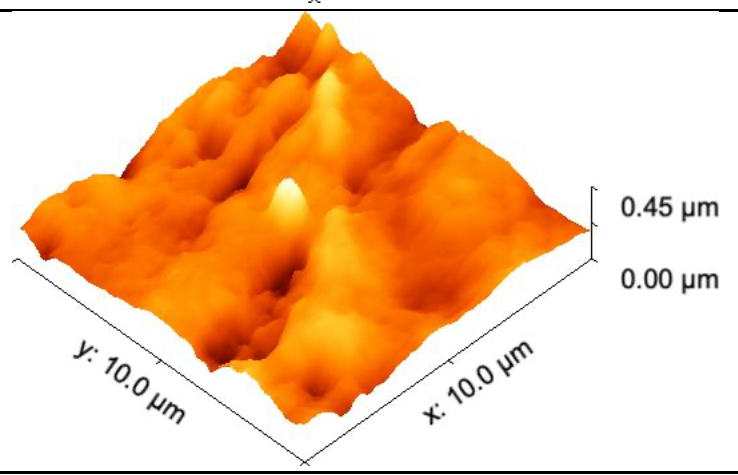	46.20 ± 10.14	63.87 ± 15.21	1.0296 ± 0.013

7. On one hand, the authors report the contact angle of tetradecane on PMMA as approximately 0° in Fig S2 (a), but in Fig. S2 (b) the contact angle of water on PMMA is reported as 71.22 ± 3.88°. The author should maintain uniformity (in terms of significant digits) in reporting experimental values. Is it true that the authors can resolve optical contact angle with second decimal place resolution, hard to believe.

We agree with the reviewer about the lack of consistency in the previous version of the paper. The tetradecane droplet on PMMA exhibits near complete wetting in air and is therefore reported as ~0°. Contact angles were determined from side-view images, processed using the LBADSA method, with standard deviation obtained by averaging over three repeats. We have revised the water

contact angle on PMMA to 71.2 ± 3.9° to remove the unrealistically stated level precision: "tetradecane exhibits near-complete wetting on PMMA in air (contact angle ~ 0°) while water droplets show a significantly higher contact angle of 71.2 ± 3.9° (see supplementary section SM 4 for contact angle measurements)." In Supplementary Material, section SM 6, all contact angle values are now reported with one decimal place for uniformity.

8. On Page 2 last line, the authors write, "The simulated evolutions of the interface configuration agree well with experiments, as shown in Fig. 1(b-c)." It is known that the Surface Evolver provides equilibrium configuration as a result of energy minimization. So how can the authors investigate the "evolutions of the interface configuration", as it is a dynamic process.

By "evolution" we referred to the change in interfacial configuration as more working liquid is added. At each step, we perform energy minimisation using Surface Evolver to compute the equilibrium configuration. The sequence of these equilibrium states therefore captures the quasi-static progression of interface shapes during capillary lifting. The reviewer's comment made us realise that using the term in this context could lead to some confusion. To improve the clarity, we revised the main text "This experimental setup enables us to capture the progressive changes in the bridge's shape as the working liquid rises, including the deformations and potential rupture which ultimately leads to distinct dewetting outcomes."

9. On Page 5, second paragraph, the authors write, "When a droplet forms a capillary bridge between the solid and the air interface,....". How can a droplet form capillary bridge between a solid and air interface, as at the air side the droplet will form curved interface due to surface tension. To form a capillary bridge, a droplet should have two interfaces on both side. Again it is not clear due to poor experimental description.

We thank the reviewer for this question. We believe the revised experimental description in response to Question 1 should now clarify this point. Specifically, in the revised manuscript we now clarify the use the term capillary bridge: "As the working fluid rises, the droplet forms a capillary bridge connecting the bottom substrate and the top air–droplet interface, while being laterally surrounded by the working fluid. This resembles classical capillary bridges between parallel solid plates, except that here the air interface acts as a deformable soft surface; we therefore refer to this structure as a capillary bridge." As the reviewer correctly notes, the top droplet–gas interface is curved due to surface tension, also shown in Fig. 1c. We use the term 'capillary bridge' because the droplet connects two boundaries (the solid substrate below and the gas interface above), similar to classical capillary bridges between parallel plates. The key difference is that in our system, the upper boundary is a deformable fluid interface rather than a rigid solid surface.

Reviewer #2 (Remarks to the Author):

Report on the manuscript "Droplet Removal by Capillary Lifting" by Kae Sun et al.

This manuscript studies the removal of liquid droplets from surfaces using an immiscible working liquid.

I read the article twice and did not fully understand it. The figures are unclear (e.g., the formation of the gas layer in Figure 1). Much information is missing. The key parameter governing the physical process of droplet removal is the force of gravity. The article does not specify the density of the liquid. How can readers understand this article? In my opinion, the roughness of the solid surface should be indicated, as it can affect the attachment of the droplet contact line to the surface.

I do not recommend this article for publication in the present form.

We thank the reviewer for their comments and agree with them that the presentation was unclear in several places. We have now extensively revised the manuscript and enhanced the description of the experimental process, results, rationale, and the figures throughout. We believe the revised manuscript is significantly better, with improved readability, narrative, and clarity.

What the reviewer referred to as the 'gas layer' is the top droplet-gas interface. The capillary bridge in our experiments is laterally surrounded by the working fluid, while its top is directly exposed to air, resulting in a droplet–gas interface. The text describing the experimental setup has been fully revised to provide a better description. The new Fig. 1 now includes a second camera view that captures the air interface, allowing clear visualisation of the droplet–gas and liquid–gas interfaces. We have also added a new Supplementary Material section SM 1 that fully documents the changes of the droplet/bridge shape during the capillary lift-off using the dual-camera system to illustrate the interfacial configurations.

We have also added density values in the revised Supplementary Material, section SM 2. In our system, the Bond number is close to unity (Bo ~ 0.2–1.4, see revised Supplementary Material, section SM 3), indicating that both capillary forces and gravity should be considered, something implemented by our modelling. We would only expect the system to be governed solely by gravity if the Bond number were >> 1, which is not the regime studied in this work.

We thank the reviewer for the suggestion to include substrate roughness characterization, something also recommended by reviewer 1. This has now been addressed using atomic force microscopy measurements in the newly added Supplementary Material, section SM 6, along with contact angle hysteresis measurements.

Reviewer #3 (Remarks to the Author):

In this work, droplet removal from solid surfaces was studied by immersing the droplets in a working fluid without surfactants. A fluidic device was employed to quasi-statically raise the working liquid, thereby creating a liquid bridge (surrounded by the working fluid) between the air interface and the solid surface, which facilitated the removal of sessile droplets. Experiments were conducted on a range of substrates, with variations in both the working liquids and the fluids forming the droplets. The process was also analyzed theoretically using Surface Evolver under quasi-static conditions, accounting for interfacial energies and gravity. A wetting-dewetting diagram was obtained based on the two characteristic contact angles of the liquid bridge confined between the two heterogeneous interfaces: cosθdl (liquid-droplet-solid) and cosθt (liquid-droplet-air). Dewetting was found to be favored when cosθdl < cosθt. The dewetting method of capillary lifting was subsequently compared with detergent soaking experiments, and was found to provide higher removal efficiency. Although this study systematically demonstrates the mechanism of droplet removal by capillary lifting through experiments supported by Surface Evolver simulations, the wetting–dewetting phenomena may also be interpreted through a thermodynamic analysis of spreading coefficients (cosθdl ~ -1). Furthermore, if the raising rate of the working fluid is sufficiently high, a transient liquid bridge may not form, and the dewetting process instead corresponds to the contact angle increasing to 180°. The authors should examine whether the raising rate influences the wetting-dewetting diagram. If it does not, the capillary lifting method can be regarded as thermodynamically driven, and the liquid bridge plays no significant role; otherwise, it is kinetically driven, and the formation of the liquid bridge becomes important. Since the overall contribution of the work is not yet clear, I cannot recommend this manuscript for publication in its current form (rejection is suggested, with resubmission recommended after major revision). Nevertheless, the manuscript would benefit from addressing the following points to improve clarity and strengthen its presentation.

We thank the reviewer for this insightful comment regarding the thermodynamic versus kinetic nature of the dewetting process. This has prompted us to conduct more experiments (detailed further down) and has helped us improve the manuscript. The reviewer suggests that the spreading coefficient, with $\cos\theta_{dl} \approx -1$, determines whether a droplet favours dewetting. While the spreading coefficient can indicate wetting behaviour in a simpler binary system, it does not fully capture the physics of capillary lifting. In our system, the droplet is confined in a capillary bridge between two interfaces: the solid substrate below and the deformable air interface above. Both interfaces must be accounted for in the wettability analysis. The capillary bridge imposes a geometric constraint that modifies the energy landscape compared to simple spreading on a flat surface.

To address the reviewer's concern regarding the influence of the raising rate, we have systematically evaluated the effect of injection velocity on dewetting (newly added Fig. 2). We varied the velocity and, correspondingly, the capillary number *Ca*, and observed that dewetting outcomes remain consistent for $Ca < 1.3 \times 10^{-7}$. Beyond this threshold, dewetting efficiency decreases with increasing *Ca*, indicating the onset of dynamic effects. All experiments in this work were conducted at $Ca \sim 10^{-8}$, well within the quasi-equilibrium limit, confirming that capillary lifting, as explored in this work, is fundamentally thermodynamically driven. See details in Fig. 2 and associated text: "Before discussing the physics implications of the results shown in Fig. 1c–d, we note the excellent agreement between the experimental measurements and equilibrium computer simulations. This confirms the predictive power of simulations, provided the capillary rise occurs quasi-statically and thermodynamic equilibrium is well approximated at all times. Experimentally, this is not obvious

despite the low rising speeds. We therefore decided to explore systematically the experimental window where these conditions are met (Fig. 2). For rising velocities below $5 \times 10^{-6}\ m/s$ (capillary number $Ca \leq\ 1 \times 10^{-7}$) , the dewetting percentage remains essentially unchanged, indicating that the system reaches a quasi-static equilibrium. For velocities above $5 \times 10^{-6}\ m/s$ ($Ca > 1 \times 10^{-7}$), the dewetting percentage decreases with increasing velocity, demonstrating that the system enters a kinetic regime. We therefore ensured quasi-static experimental measurements throughout the study (data point highlighted in red in Fig. 2).

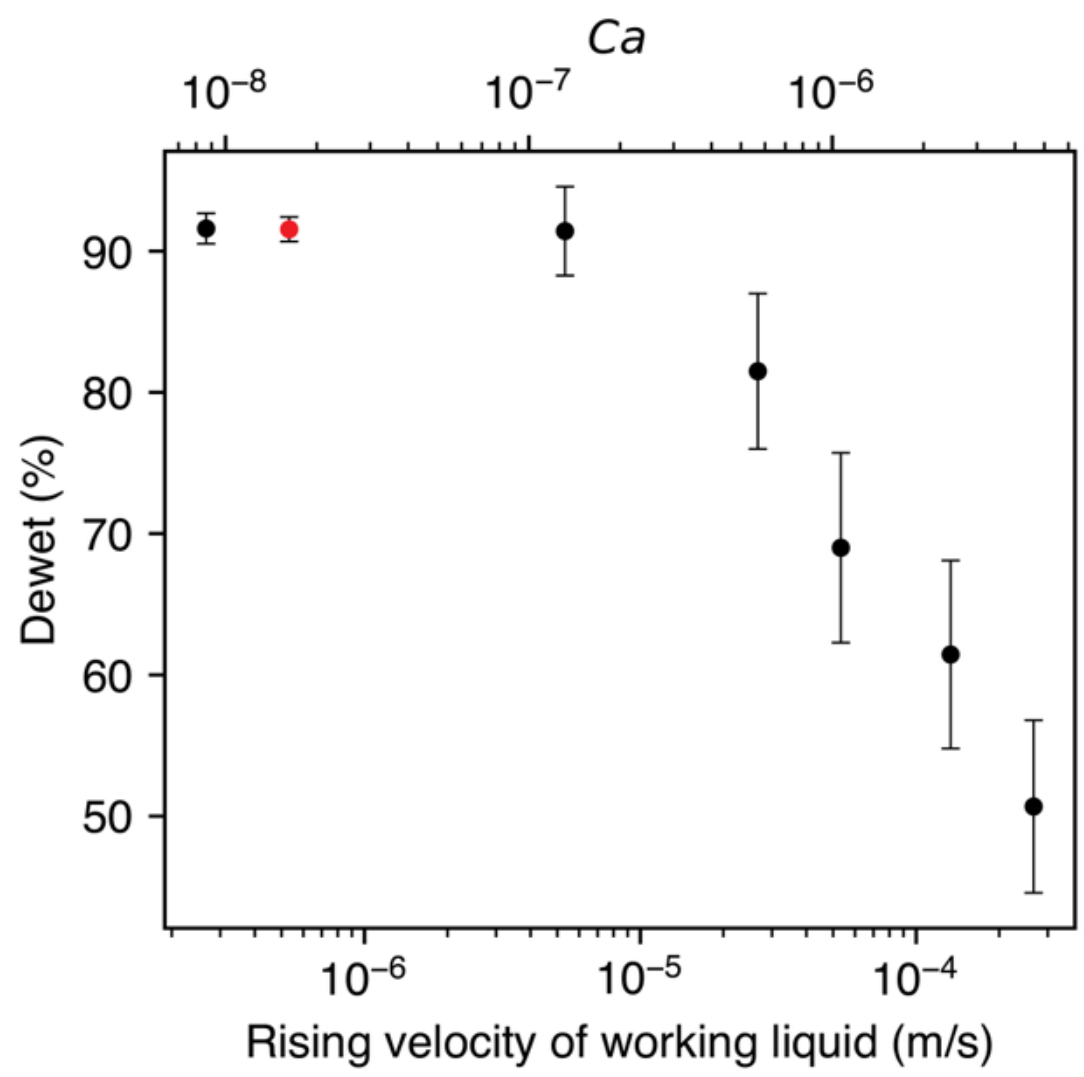

Figure 2. Capillary lifting at different rising velocities of the working liquid. A tetradecane droplet on PMMA is lifted by injecting water at velocities ranging from 10^{-7} to $10^{-4}\ m/s$. The dewetting outcome changes from nearly complete detachment at lower velocities to partial detachment at the higher velocities. The data point highlighted in red corresponds to the dewetting velocity employed in this work (unless specified otherwise). These velocities correspond to capillary numbers in the range $Ca \sim 10^{-9} - 10^{-6}$.”

Importantly, being in the quasi-equilibrium regime does not imply that the capillary bridge is insignificant. On the contrary, the bridge is essential for overcoming the adhesion of the droplet to the substrate. Without the formation of the capillary bridge, droplets would remain bound indefinitely, as demonstrated in our soaking experiments (Fig. 5 and the newly added supplementary material, section SM 11). Thus, the capillary bridge provides the geometric and energetic pathway that allows the system to transition from an adherent sessile droplet to detachment under quasi-static conditions.

1. The manuscript focuses on the contact angle of the droplet in liquid (θdl) and the effective top contact angle (θt) in establishing the dewetting criterion. However, during the actual removal process, the sessile droplet contact angle is expected to increase gradually as the working liquid rises. Reporting such dynamic contact angle data would strengthen the interpretation of the dewetting mechanism.

We agree that the droplet bridge configuration changes gradually during capillary lifting as the capillary bridge is stretched and eventually ruptures. Correspondingly the droplet contact angle also varies. However, for understanding the capillary lifting mechanism the most relevant contact angle is

the receding contact angle of the droplet on the surface. In a dewetting case (such as Fig. 1c), we observe that the bottom contact angle varies within approximately 5 degrees from the first well-defined capillary bridge to the last bridge configuration before rupture. Significantly, taking the measured receding contact angle as input in our simulations gives a good agreement between experimental and simulation morphologies.

That being said, we do recognize the need to better characterise the surface roughness and contact angle hysteresis for the combination of fluids and the choice of substrates we use in this work. This data is now provided in the newly added Supplementary Material, section SM 6 "Substrates roughness and contact angle hysteresis:

"As described in the main text, we used commercially available substrates to validate the effect of capillary lifting in practical cleaning. These surfaces are unlikely to be ideal, exhibiting both topographic and chemical heterogeneities. Here, we characterised the substrates using contact angle hysteresis (CAH) measurements and atomic force microscopy (AFM) imaging.

In the CAH measurements, droplets were deposited on the substrates while immersed in the working liquid, and the droplet was injected and withdrawn obtain advancing and receding angles (Table II). Measurements under liquid are inherently much more challenging than measurements in air due to droplet mobility upon needle contact, non-uniform pinning along the contact line, gravitational deformation, and substrate heterogeneity across different measurement locations. These factors contribute to the relatively large uncertainties. In Table III, the advancing and receding angles were determined directly from video frames at the moments when the contact line was advancing or receding, with standard deviations calculated from three repeated measurements.

For the capillary bridge simulations, we used the receding contact angle observed from the corresponding capillary bridge retraction experiment. The values lie within the range of the experimentally measured CAH for the droplet setup (as reported in Table II). This means the measurements remain sufficiently reliable for estimating the relevant contact angles, which is further supported by the good agreement between the experimental and simulated capillary bridge morphologies.

TABLE III. Advancing and receding contact angle measurements in the surrounding liquid. All measurements were repeated at least three times to determine the standard deviation.

Surface	Droplet composition	Surrounding liquid	Advancing angle ± standard dev. (°)	Receding angle ± standard dev. (°)
Stainless Steel	Tetradecane	Water	118.2 ± 6.5	37.0 ± 7.1
Stainless Steel	Decane	Water	125.1 ± 6.0	53.4 ± 5.8
Stainless Steel	Tetradecane	Formulation	118.7 ± 3.9	30.8 ± 1.4
Glass	Decane	Water	132.6 ± 5.6	65.8 ± 1.3
Glass	Tetradecane	Water	132.2 ± 5.5	57.9 ± 1.4
Glass	Silicone oil	Water	129.2 ± 6.5	118.7 ± 4.9
Glass	Water (fluorescein dyed)	Tetradecane	81.2 ± 8.2	75.5 ± 11.3
PTFE	Tetradecane	Water	28.2 ± 3.4	13.0 ± 2.0

PTFE	Tetradecane	Formulation	32.0 ± 9.8	25.0 ± 4.8
PTFE	Decane	Water	26.0 ± 3.6	15.2 ± 2.0
PTFE	Decane	Formulation	65.1 ± 4.4	40.4 ± 2.8
PMMA	Water (fluorescein dyed)	Tetradecane	98.6 ± 8.9	88.5 ± 3.1
PMMA	Tetradecane	Formulation	106 ± 20.3	130.0 ± 4.6
PMMA	Tetradecane	Water	126.5 ± 7.0	55.6 ± 8.1
PMMA	Squalane	Water	119.6 ± 3.8	59.6 ± 6.0
PMMA	Olive oil	Water	113.0 ± 16.8	50.5 ± 6.0
PMMA	Decane	Water	89.1 ± 6.1	59.6 ± 4.4
PMMA	Tetradecane (Sudan IV dyed)	Water	81.1 ± 11.1	71.7 ± 12.2
PC	Tetradecane	Formulation	113.6 ± 9.4	50.2 ± 4.3
PC	Tetradecane	Water	73.9 ± 8.9	19.9 ± 3.7
PC	Decane	Water	78.0 ± 2.0	22.2 ± 5.1

AFM imaging was performed in contact mode using a JPK NanoWizard 3 (Bruker, USA) at ambient temperature and humidity. The AFM tip/cantilever (SNL-10 tip A, Bruker AFM Probes, USA, nominal spring constant 0.4 N/m) was calibrated using its thermal spectrum after the measurements. Representative AFM images and the corresponding roughness parameters (average ± standard deviation) were obtained from three repeated measurements across randomly selected areas of each sample. Roughness statistics were processed using Gwyddion[5] (Table IV).

TABLE IV. AFM topographic images of the substrates and corresponding roughness parameters. Each measurement was repeated at least three times to determine the standard deviation.

Material	AFM Topographic image	Mean roughness (Sa) in nm	RMS roughness (Sq) in nm	Surface area/projected area (r)
Stainless steel	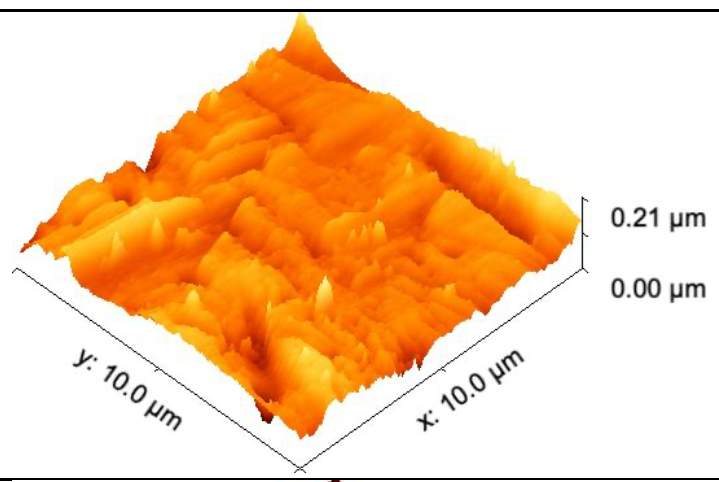	24.13 ± 13.38	33.37 ± 21.10	1.032 ± 0.030
Glass	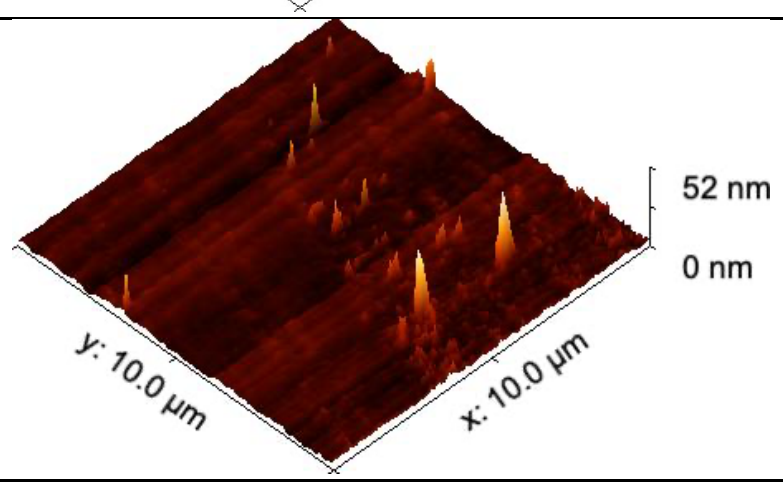	1.26 ± 0.39	1.71 ± 0.65	1.0004 ± 0.001

PMMA	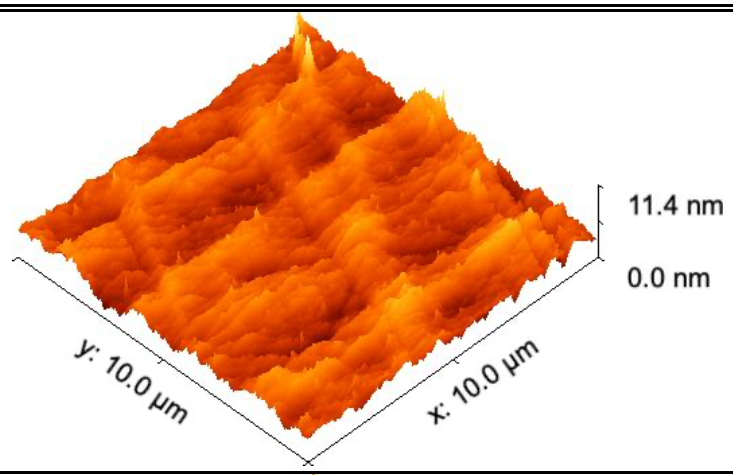	0.97 ± 0.18	1.62 ± 0.60	1.000 ± 0.001
PC	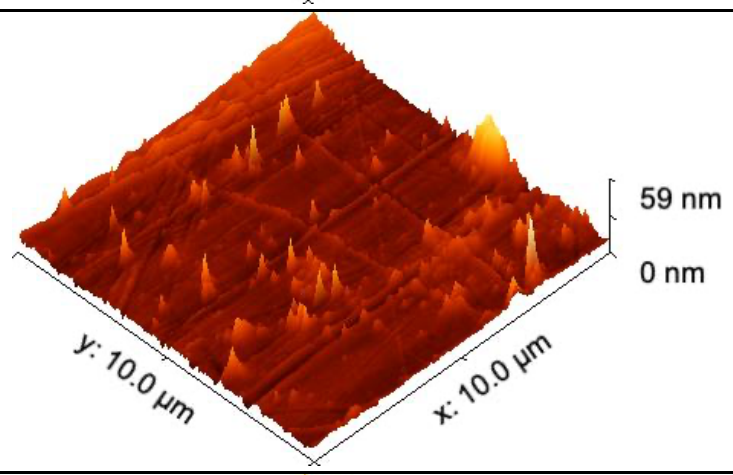	2.44 ± 0.58	4.21 ± 1.29	1.003 ± 0.002
PTFE	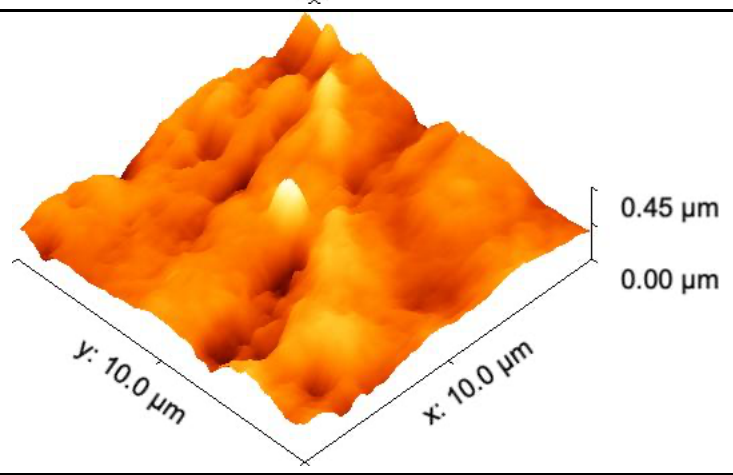	46.20 ± 10.14	63.87 ± 15.21	1.0296 ± 0.013

2. For the wetting scenario (in contrast to the dewetting case), the time evolution of the dynamic contact angle should be reported as the working liquid rises. Presumably, the contact angle will eventually reach a constant value.

In the wetting case (Fig. 1d), the contact angle varies by only approximately 3 degrees during the rise of the working fluid. Such small changes are within the uncertainty arising from contact line pinning and measurement error. This minimal variation is likely due to the favourable wetting of the droplet to the bottom substrate; the rising working liquid–gas interface cannot significantly alter the droplet–liquid interface profile. In contrast to the dewetting case where the droplet undergoes substantial deformation and eventually detaches, in the non-dewetting (wetting) case the droplet essentially maintains its profile throughout the process (Fig. 1d).

As noted in response to the Question 1, the contact angle behaviour depends on the specific combination of fluid pairs and substrate properties. The characterisation of surface properties and contact angle hysteresis is provided in Supplementary Material, section SM 6.

3. The manuscript does not present a systematic study of capillary lifting using aqueous surfactant solutions at different concentrations, but instead relies on a low-concentration glycol ether formulation to tune interfacial tensions. Employing the same working fluids in both soaking and capillary lifting experiments would allow for a more meaningful comparison between the two approaches.

We thank the reviewer for this helpful suggestion. We have now included the soaking experiments corresponding to the three systems discussed in the manuscript. These results are provided in the newly added Supplementary Material, section SM 11: "Soaking experiments for systems shown in main text

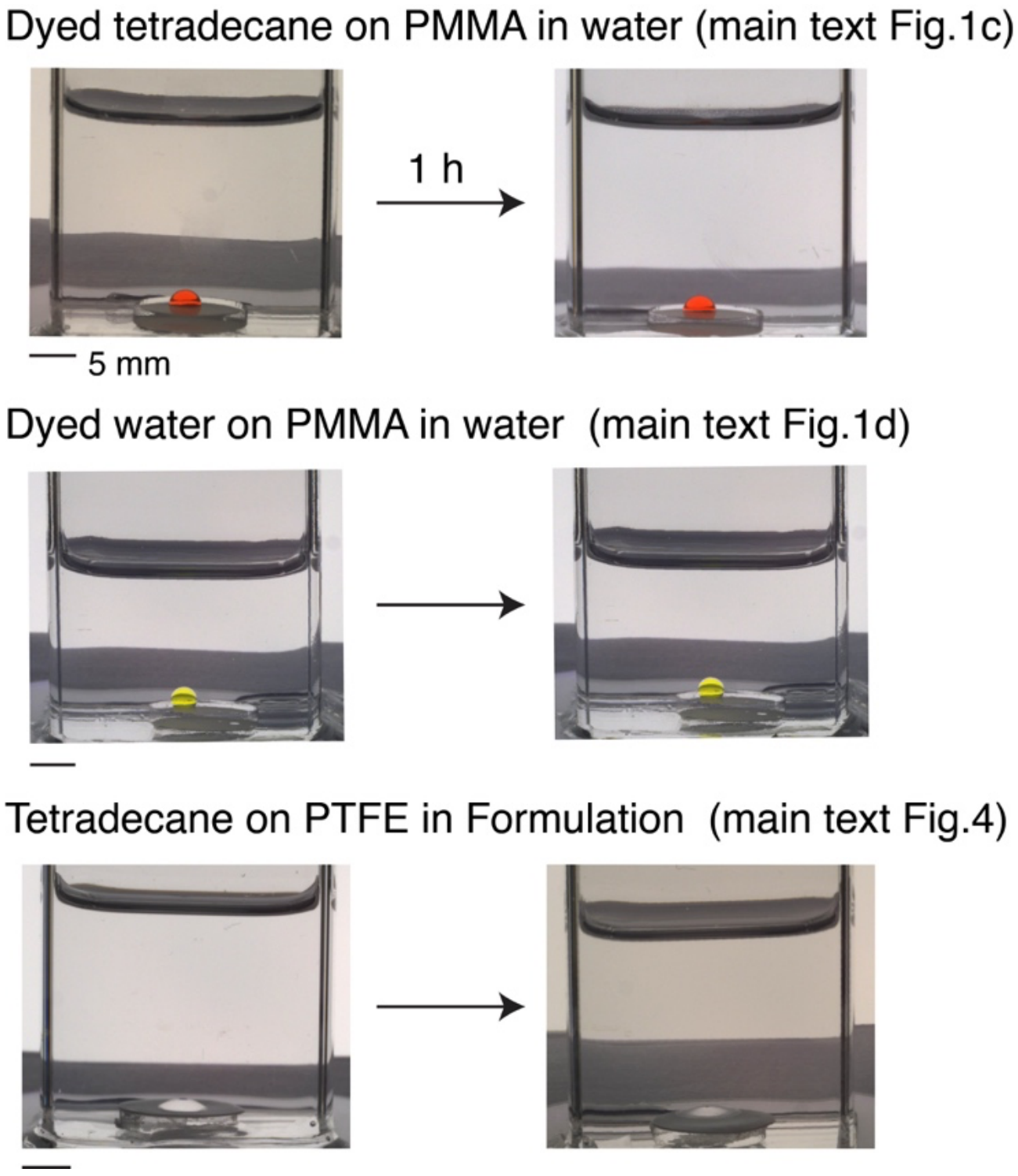

Fig. S8. Droplet soaking experiments for the systems shown in Fig. 1 and Fig. 4 of the main text. A 10 μL olive-oil droplet was deposited on the corresponding substrate at the bottom of a bath of the surrounding fluid. The system was left undisturbed for 1 h, and the droplet remained in place, showing no detachment."

In the main text, we noted these reference soaking experiments in the Results section to highlight why the capillary lifting process is essential for achieving droplet removal. "…For consistency, we also performed soaking tests for the systems shown in Fig. 1 and Fig. 4, and similarly observed no detachment under static immersion (see details in supplementary section SM 11)…"

4. The study employs a quasi-static lifting process, but no comparison is provided for different lifting speeds (faster vs. slower rise). Such tests would help distinguish whether the observed dewetting is governed primarily by quasi-static thermodynamic equilibrium or whether kinetic effects significantly influence the outcome.

This important point is closely related with the earlier comment before Question 1. We therefore refer the reviewer to our response to this first comment where we discuss the quasi-static nature of our experiments and show the outcome of our additional experiments at various lifting velocities (new Fig. 2 and associated text).

5. I suggest that the authors clarify whether a simple bulk immersion test, in which the droplet is directly deposited through the working liquid bath onto the solid surface, yields the same equilibrium configuration as observed in the wetting or dewetting scenario. Such a check would help determine whether the mechanism is fundamentally thermodynamic in nature or dependent on the kinetic lifting process.

We have now conducted these tests as part of our response to the Question 3. The top two panels of the newly added Fig. S8 show soaking experiments corresponding to both the dewetting and non-dewetting systems tested in the main text. In both cases, the droplets remain attached to the bottom substrate after prolonged soaking.

Combined with the data presented in the new Fig. 2 in the main text, we believe the process studied in this paper is thermodynamically driven. The capillary force is necessary to overcome the adhesion energy that binds the droplet to the substrate.

6. The manuscript relies primarily on 10 µL droplets, where the capillary length is comparable to the droplet size. As shown in Figure S3, increasing droplet size leads to higher dewetting percentages due to gravitational effects. Since droplet size affects the balance between capillary and gravitational forces, it would be important to discuss whether the applicability of dewetting phenomena extends across a broader range of droplet volumes.

We thank the reviewer for this insightful comment on the role of droplet size and the balance between gravity and capillary forces. As the reviewer noted, we show in Fig. S4 of the revised manuscript that when the droplet has a lower density than the surrounding liquid, the dewetting percentage increases with droplet size due to the stronger impact of gravity.

The Bond number is a dimensionless parameter that characterises the relative importance of gravitational versus capillary forces. For the cases where the working fluid is pure (i.e., the dewetting and non-dewetting cases discussed in Fig. 1c and 1d), the Bond number is in the range of 0.2 to 0.4, indicating that capillary forces dominate over gravitational forces. However, since these values are not far from unity, gravity still contributes and must be accounted for in the analysis (similar reasoning applies to the capillary length scale). When the working fluid is the formulation liquid, the interfacial tension between the droplet and liquid decreases, resulting in a Bond number of approximately 1.3, slightly above unity. This indicates that gravitational effects become stronger in this case. Nonetheless, across all our experimental systems, the Bond number remains comparable to unity ($0.2 \leq Bo \leq 1.4$), meaning that both gravity and capillary forces make significant contributions to the dewetting phenomena.

In the regime where $Bo \gg 1$, we would expect gravity to govern the physics of these ternary systems. For example, if the droplets were much larger than those in our current study (representing a significant increase in the characteristic length scale L), the removal outcome would depend primarily on the density difference between the droplet and the surrounding liquids, with capillary effects becoming secondary. Our work therefore focuses on the practically relevant regime where capillary and gravitational forces are both important and work together to facilitate droplet removal.

To reflect these comments, we added to supplementary material, section SM 3 "The capillary length of all the systems in this work ranges from 1.87 to 4.94 mm and the droplet volume is 10 μL, making the capillary length comparable in magnitude to the droplet size. This can be rationalised by noting that the Bond number $Bo = \Delta\rho g L^2/\gamma$ ($\Delta\rho$ is the density difference between the droplet and the working liquid, g is the gravitational acceleration L is the characteristic droplet length, and γ is the droplet–liquid interfacial tension) being close to unity in this work, typically 0.2 < *Bo* < 1.4.

[...]

These observations highlight the necessity of considering gravitational effects when *Bo* ~ 1, which have been incorporated into our computational model. For much larger droplets where *Bo* >> 1, gravity would be expected to govern the ternary fluid system, and droplet detachment would be determined primarily by the density difference between the droplets and the working liquid. Such gravity-dominated regimes are beyond the scope of this work, which focuses on the capillary lifting phenomena."

Furthermore, we carried out simulations in the zero-gravity limit ($Bo \rightarrow 0$), keeping all other parameters identical to those in the dewetting case Fig. 1c. As shown in Fig. R1, the droplet still dewets. This confirms that capillary lifting can operate without gravitational assistance.

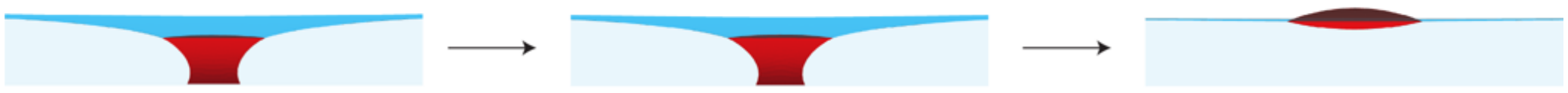

Figure R1: Simulation sequence corresponding to Fig. 1c of the main text, but performed without gravity ($Bo \rightarrow 0$). From left to right, the working liquid rises. The droplet still undergoes capillary driven dewetting and detachment.

7. In the Surface Evolver simulations, is the solid-liquid interfacial tension (or cos θ) obtained directly from the same experiments rather than determined independently? If so, does this approach yield the correct solid-liquid tension for the dewetting case?

The contact angle cos θ was obtained directly from the capillary bridge configuration in the experiments and used as input to the Surface Evolver simulations. As discussed in our response to Question 1, the contact angle exhibits only small variations (≤ 5 degrees) during the bridge elongation process. The fact that the simulations closely match the experimental observations (Fig. 1c) indicates that these input values are appropriate for describing the capillary lifting phenomena.

Nonetheless, to validate this approach, we have also characterized the contact angle hysteresis through independent methods using advancing and receding angle measurements of sessile droplets immersed in the working fluid. These data are presented in the newly added Supplementary Material, section SM 6. The contact angle values used in our simulations fall within the range of the measured hysteresis data, confirming that our input parameters are physically reasonable.

8. The formation of the unstable liquid bridge is indeed interesting; however, it is inherently coupled with the rising working fluid due to the presence of another air–liquid interface. This process may provide a pathway to overcome the kinetic barrier, which should be explained in greater detail. In

addition, regime V of Figure 2f cannot be explained solely by free-energy minimization and should likewise be discussed in more detail.

We thank the reviewer for this comment. Following the reviewer's earlier suggestions, we have now tested the velocity dependency on the dewetting outcome in the newly added Fig. 2. The results show that our experimental velocity is well within the quasi-static regime ($Ca \sim 10^{-8}$, with consistent behaviour up to $Ca < 1.3 \times 10^{-7}$), confirming that the process is thermodynamically driven rather than kinetically limited. In addition, the newly added soaking experiments (Fig. S8, addressing Questions 3 and 5) show that droplets remain adhered to the substrate without the capillary bridge. This demonstrates that capillary lifting provides a necessary geometric and energetic pathway for droplet removal.

Regime V refers to partial dewetting where the detached portion floats as a liquid lens while a sessile droplet remains at the bottom. To capture this in simulations, we added working liquid incrementally and performed energy minimization at each stage. For Regime V, we observe that the bridge thins in the middle while experiencing spreading at the top and bottom interfaces. Eventually, the capillary bridge splits into two separate bodies. Conceptually, in the quasi-static limit, the capillary bridge shape is a solution of the Young-Laplace equation (with gravity). The breaking of the neck corresponds to the loss of solution to the Young-Laplace equation. Similar behaviour has been reported in other studies, such as that by Sadullah et al. (Commun. Phys. 7, 89, 2024) for gravity-driven partial droplet detachment.

It is worth noting that partial dewetting occurs within a narrow window of interfacial parameters in the simulations (Figs. 3f, 3g, and the updated supplementary section SM 7). In the experiments, partial dewetting is more prominent due to surface roughness and chemical heterogeneity.

Following the results of the new Fig. 2, we see that the fraction of droplet dewetting depends on the working fluid rising velocity for $Ca > 1.3 \times 10^{-7}$. For future work it is indeed interesting to systematically explore kinetic effects during the capillary lifting process. However, it is beyond the scope of the present paper that focuses on small Ca (quasi-static limit). We add in the conclusion:

"… It is also interesting to systematically investigate kinetic effects during the capillary lifting process as the velocity of the rising working fluid is increased."

We thank the reviewer for their comments. We have addressed the points raised and made specific changes as detailed below.

For the sake of clarity, the comments are shown in black, our response appears in blue and any resulting change in the manuscript is shown in red.

Reviewers' comments:

Reviewer #3 (Remarks to the Author):

The authors have adequately addressed my previous query by claiming that the capillary lifting mechanism is thermodynamically driven. However, this is achieved by deliberately restricting the experimental setup to a strictly quasi-static limit with an ultra-slow fluid raising velocity of about 5×10-7 m/s. Furthermore, as the revised manuscript indicates, the dewetting efficiency decreases significantly when the velocity increases and the system enters a kinetic regime. The sharp decline in efficiency under dynamic conditions represents a fundamental flaw in the manuscript's core premise.

Since a true thermodynamic transition should be path-independent (and thus independent of the lifting velocity), the observed rate-dependency suggests that the underlying mechanism is kinetically limited rather than purely thermodynamic. This indicates that the proposed "capillary lifting" is not a robust removal mechanism, but a metastable state that is easily disrupted. As velocity increases, hydrodynamic effects likely force the capillary bridge to rupture prematurely, preventing complete dewetting. Furthermore, the requirement for an exceptionally slow and precisely controlled displacement rate to maintain these quasi-static conditions poses significant practical challenges. Given this lack of robustness under dynamic conditions and the resulting limited technological relevance, I cannot recommend the manuscript for publication in its current form.

We thank the reviewer for the comment. However, we disagree that the observed velocity dependence is a flaw in the manuscript's core premise. Rather, it reflects the expected transition from a thermodynamically governed regime to a kinetically limited one. As shown in Fig. 2 of the paper, there are two regimes. At low velocities (small capillary number Ca), the capillary lifting transition is thermodynamically driven, meaning it only depends on the energetics of the interfacial free energies and there is no velocity dependence. In this regime, the sequence of configurations (the trajectory) is essentially the same as the droplet dewets. With increasing velocity ($Ca \gtrsim 10^{-7}$), indeed hydrodynamic effects become more important. The balance of thermodynamic driving force and viscous dissipative force affects the dewetting trajectory.

Many well-established processes and techniques rely on thermodynamically driven capillary effects in the same low *Ca* regime as used here in order to achieve high consistency and precision:

1. Capillary peeling of thin hydrophobic films

Khodaparast et al. [*Phys. Rev. Lett.* **119**, 154502 (2017)] showed that the removal rate remains near 100% up to a critical speed and then decreases sharply at higher velocities. For a 90 nm film, this threshold corresponds to $Ca{\sim}10^{-7}$. Beyond this point, the dissipated energy from viscous effects exceeds the adhesion energy, and the liquid flows over the film without initiating detachment.

2. Colloidal self-assembly

Manoharan et al. [*Science* **301**, 483–487 (2003)] experimentally showed that robust capillary driven colloidal self-assembly (e.g., to form desired symmetric colloidal clusters) requires sufficiently low evaporation rates to avoid kinetic traps, typically $Ca{\sim}10^{-9}$. Theoretical work by Lauga and Brenner [*Phys. Rev. Lett.* **93**, 238301 (2004)] confirmed that the quasi-static limit is essential: at higher velocities, viscous forces prevent particles from packing into their symmetric, energy-minimized configurations.

3. Evaporation-driven aggregation of elastic objects

Hadjittofis et al. [*Journal of Fluid Mechanics* **792**, 168–185 (2016)] showed that consistent morphologies corresponding to the global energy minimum can be achieved only in the quasi-static limit (e.g. $Ca{\sim}10^{-9}$ in aqueous system). At higher velocity, the final pattern is dictated by kinetics rather than capillary-driven thermodynamics.

Such low-Ca operation has further been harnessed in numerous applications, including meniscus-guided coating of organic electronics [*Nat Commun* **9**, 534 (2018)], capillary-driven microfluidics [*Journal of Fluid Mechanics* **852**, 60–104 (2018)], and highly complex colloidal self-assembly [*PNAS* **121**, e2403690121 (2024)]. These technologies deliberately operate in this low-velocity regime because higher speeds introduce kinetic effects that suppress the capillary mechanisms on which they rely. Our study situates itself within similar operating window. We acknowledge the reviewer's point that operating in a thermodynamically dominated regime requires slow velocities, and we consider investigating higher *Ca*, where kinetic limitations emerge, to be an important direction for future work.

To address this point, we have made several changes to the manuscript. First, we now include a discussion about the fact that many established capillary-driven techniques such as the peeling hydrophobic films, colloidal self-assembly and aggregation of elastic objects, necessarily operate at low *Ca*.

"A similar transition from a thermodynamically dominated to a kinetic regime occurs in numerous capillary-driven phenomena where operation in the low-*Ca* regime ensures that capillary forces dominate over viscous forces, enabling precise control over interfacial dynamics. For example, quasi-static operation is used for the peeling of hydrophobic films[22], meniscus-guided coating of organic electronics[23], aggregation of elastic objects[24], and complex colloidal self-assembly[25]. Accordingly, we maintained quasi-static experimental measurements throughout this study (data point highlighted in red in Fig. 2)."

Second, we better highlight that understanding the capillary lifting mechanism at higher Ca is an important area of future work.

"Our results show that the capillary lifting mechanism is most effective at low *Ca*, ideal for applications where a high level of control is needed or where resources are scarce. Future investigations will systematically explore the kinetic effects as the velocity of the rising working fluid is increased, including when formulated fluids containing polymers and surfactants are employed."

The authors have adequately addressed my previous query by claiming that the capillary lifting mechanism is thermodynamically driven. However, this is achieved by deliberately restricting the experimental setup to a strictly quasi-static limit with an ultra-slow fluid raising velocity of about 5×10^{-7} m/s. Furthermore, as the revised manuscript indicates, the dewetting efficiency decreases significantly when the velocity increases and the system enters a kinetic regime. The sharp decline in efficiency under dynamic conditions represents a fundamental flaw in the manuscript's core premise. Since a true thermodynamic transition should be path-independent (and thus independent of the lifting velocity), the observed rate-dependency suggests that the underlying mechanism is kinetically limited rather than purely thermodynamic. This indicates that the proposed "capillary lifting" is not a robust removal mechanism, but a metastable state that is easily disrupted. As velocity increases, hydrodynamic effects likely force the capillary bridge to rupture prematurely, preventing complete dewetting.

Furthermore, the requirement for an exceptionally slow and precisely controlled displacement rate to maintain these quasi-static conditions poses significant practical challenges. Given this lack of robustness under dynamic conditions and the resulting limited technological relevance, I cannot recommend the manuscript for publication in its current form.
