# Supplementary material for: Droplet removal by capillary lifting
Source: Commun Phys. 2026 Jun 12;9(1):307. doi: 10.1038/s42005-026-02714-4 (PMC13616733; doi:10.1038/s42005-026-02714-4)
Supplement: Supplementary file 2 [file 42005_2026_2714_MOESM2_ESM.pdf]

Supplementary Information for Droplet Removal by Capillary Lifting

Ke Sun,[1] Jack R. Panter,[2] Alvin C. M. Shek,[1] Yonas Gizaw,[3,4] Kislon Voïtchovsky,[1*] and Halim Kusumaatmaja[1,5*]

[1]Department of Physics, Durham University, Durham, DH1 3LE, United Kingdom
[2]School of Engineering, Mathematics and Physics, University of East Anglia, Norwich, NR4 7TJ, United Kingdom
[3]Whistler Center for Carbohydrate Research, Purdue University, West Lafayette, Indiana, United States of America
[4]GreenChemSolutions LLC, West Chester, Ohio, United States of America
[5]Institute for Multiscale Thermofluids, School of Engineering, The University of Edinburgh, Edinburgh, EH9 3FB, United Kingdom

[*]Correspondence to: kislon.voitchovsky@durham.ac.uk; halim.kusumaatmaja@ed.ac.uk

Supplementary Note 1: Visualising capillary lifting by dual-camera imaging

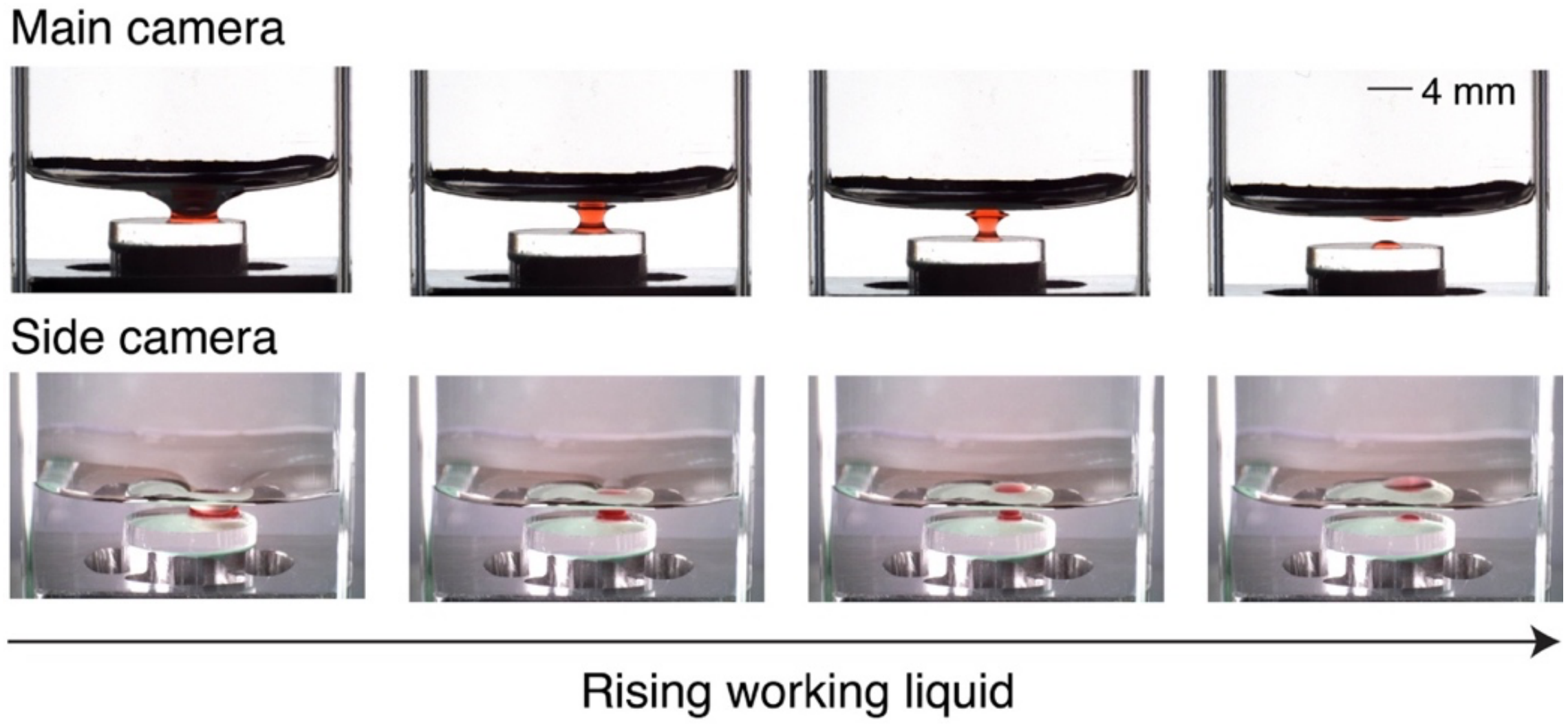

Figure S1. **The imaging system used to observe capillary lifting.** The main camera captures the full droplet configuration during lifting (top panel), while a slightly elevated side camera provides a complementary view from another angle to detect any asymmetric behaviour due to pinning (bottom panel). This side view also clearly reveals the droplet–air interface, corresponding to the top of the defined capillary bridge between air and substrate. The shown field of view of the main camera has been readjusted in each image for better visibility

Supplementary Note 2: Table of the interfacial tensions and density

The interfacial tensions of liquid–gas and liquid–liquid systems were measured using the pendant drop method. A 22G metal dispense needle (Adhesive Dispensing Ltd., Milton Keynes, UK) was employed, and visualization was carried out as described in the experimental section. Images were processed using the pendent drop plugin[1] in ImageJ freeware[2]. The measured surface and interfacial tensions are provided in Table S1. The densities are given in Table S2.

Table S1. Measured interfacial tensions.

Phase I	Phase II	Interfacial tension ± standard deviation (mN m^{-1})
Air	Tetradecane	26.6 ± 0.9
	Decane	23.6 ± 0.3
	Formulation	28.3 ± 0.3
	Olive oil	32.7 ± 0.8
	Squalane	26.1 ± 0.6
	Water (fluorescein dyed)	73.4 ± 0.9
	Tetradecane (Sudan IV dyed)	24.5 ± 0.2
	Silicone oil	19.5 ±0.3
Water	Tetradecane	45.2 ± 1.4
	Decane	36.7 ± 0.4
	Tetradecane (Sudan IV dyed)	42.3 ± 0.2
	Olive oil	35.1 ± 1.4
	Squalane	46.1 ± 1.5
	Silicone oil	35.2 ± 0.6
Tetradecane	Water (fluorescein dyed)	36.8 ± 2.8
	Formulation	18.2 ± 0.4

Table S2. Liquid densities.

Liquid	Density (kg m^{-3})
Tetradecane	762
Decane	730
Formulation	983
Olive oil	853
Squalane	810
Water (fluorescein dyed)	995
Tetradecane (Sudan IV dyed)	755
Silicone oil	930
Water	1000

Supplementary Note 3: Impact of gravitational forces

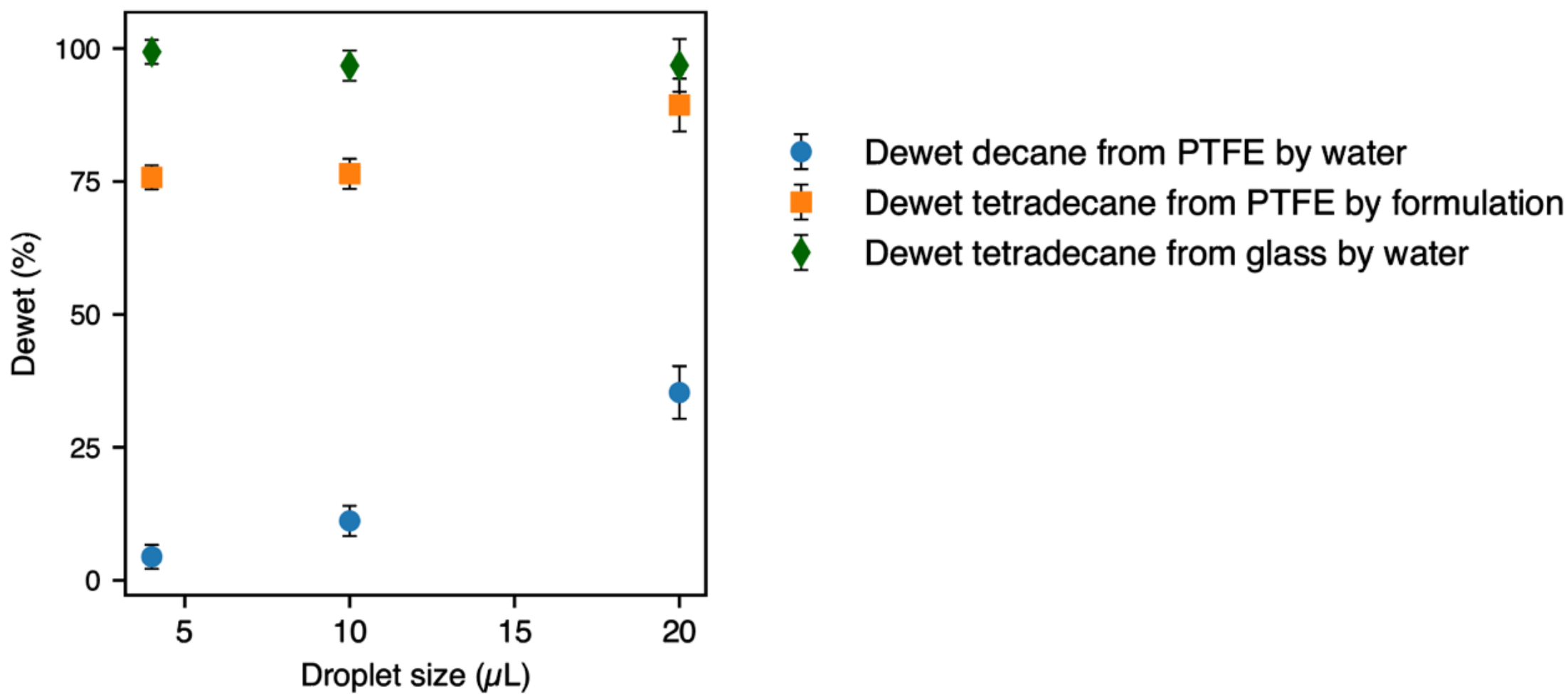

Figure S2. Influence of droplet size on dewetting behavior across different systems. The increased dewetting for larger droplets indicate that gravity cannot be neglected.

The capillary length of all the systems in this work ranges from 1.87 to 4.94 mm and the droplet volume is 10 μL, making the capillary length comparable in magnitude to the droplet size. This can be rationalised by noting that the Bond number $Bo = \Delta\rho g L^2/\gamma$ ($\Delta\rho$ is the density difference between the droplet and the working liquid, g is the gravitational acceleration L is the characteristic droplet length, and γ is the droplet–liquid interfacial tension) being close to unity in this work, typically $0.2 < Bo < 1.4$.

To assess the influence of gravitational forces, we experimentally varied the droplet size across different systems. As shown in Fig. S2, increasing the droplet size leads to a higher dewetting percentage. This trend is expected, as the droplets in these systems are less dense than the working liquid, allowing gravity to enhance dewetting for larger droplets. In systems exhibiting near complete dewetting (green, Fig. S2), the effect of droplet size becomes negligible. This is because the system is already in a fully dewetting regime therefore the contribution of gravity is minimised. These observations highlight the necessity of considering gravitational effects in the $Bo \sim 1$ regime, which have been incorporated into our computational model. For much larger droplets where $Bo >> 1$, gravity would be expected to govern the ternary fluid system, and droplet detachment would be determined primarily by the density difference between the droplets and the working liquid. Such gravity-dominated regimes are beyond the scope of this work, which focuses on the capillary lifting phenomena.

Supplementary Note 4: Contact angle measurement of tetradecane and water in air on poly(methyl methacrylate) (PMMA)

As shown in Fig. S3a, tetradecane exhibits near-complete wetting on PMMA, corresponding to a contact angle of approximately 0°. In contrast, the contact angle of a water droplet on PMMA in air is measured to be 71.2 ± 3.9° (Fig. S3b) by the LBADSA method[3].

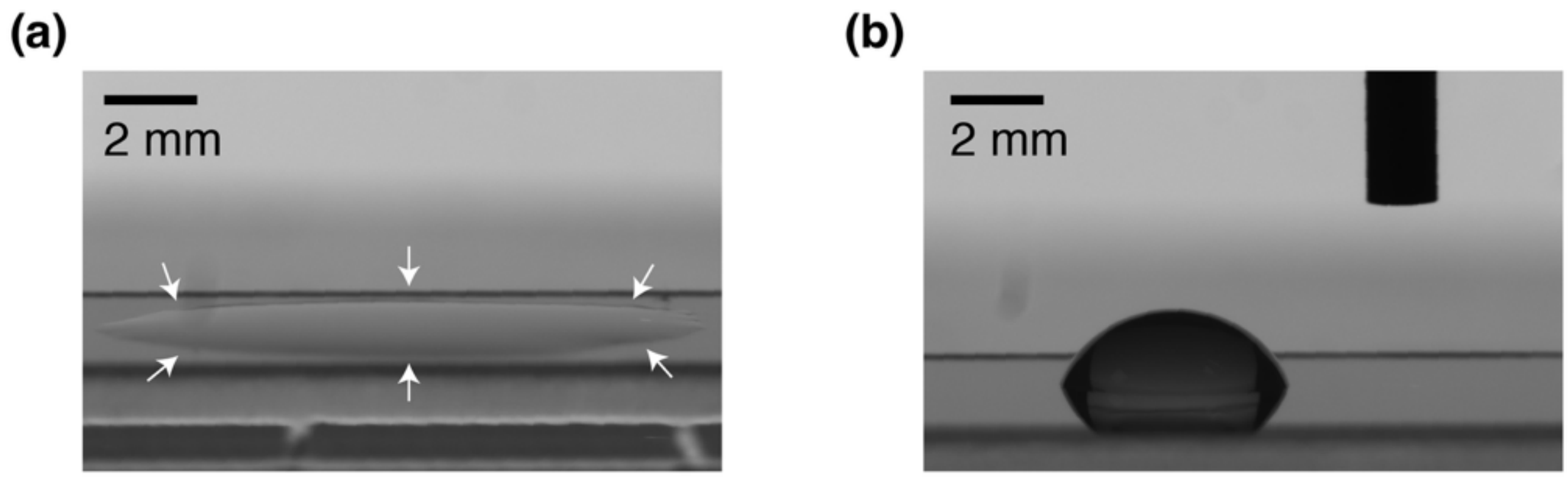

Figure S3. Contact angle measurements of 10 µL droplets on poly(methyl methacrylate) (PMMA) substrates. (a) tetradecane and (b) water. White arrows in (a) are added to aid visualisation.

Supplementary Note 5: Static contact angles of substrates and dewetting behaviour

This study employed commercially available substrates to investigate their dewetting behaviour, contributing to potential real-life applications. The selected substrates range from hydrophobic (e.g., PTFE) to hydrophilic (e.g., glass and stainless steel), covering a broad spectrum of surface wettability. The static contact angles of hydrophobic droplets on different substrates surrounded by aqueous working fluid illustrate this wettability variation (Fig. S4a). Higher static contact angles indicate weaker droplet adhesion, which leads to increased dewetting. This trend is evident across different systems (Fig. S4b), supporting the choice of surfaces used in this study.

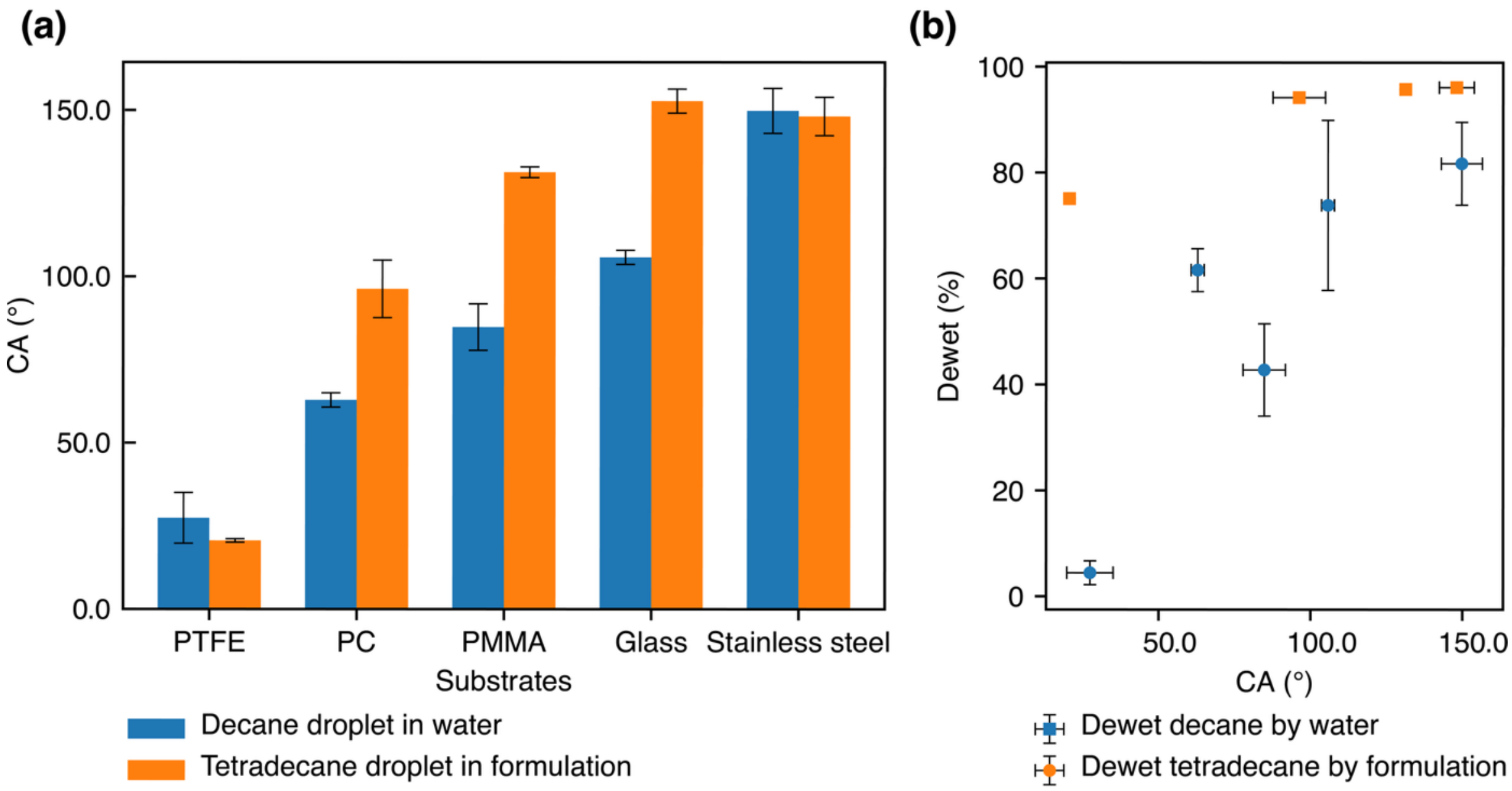

Figure S4. Measured static contact angles and dewetting outcomes for the investigated surfaces in two systems: dewetting decane by water and dewetting tetradecane by the formulation. (a) Static contact angles of droplets on various substrates surrounded by the working fluid. (b) The dewetting behaviour of droplets as a function of static contact angle.

Supplementary Note 6: Substrates roughness and contact angle hysteresis

As described in the main text, we used commercially available substrates to validate the effect of capillary lifting in practical cleaning. These surfaces are unlikely to be ideal, exhibiting both topographic and chemical heterogeneities. Here, we characterised the substrates using contact angle hysteresis (CAH) measurements and atomic force microscopy (AFM) imaging.

In the CAH measurements, droplets were deposited on the substrates while immersed in the working liquid, and the droplet was injected and withdrawn obtain advancing and receding angles (Table S3). Measurements under liquid are inherently much more challenging than measurements in air due to droplet mobility upon needle contact, non-uniform pinning along the contact line, gravitational deformation, and substrate heterogeneity across different measurement locations. These factors contribute to the relatively large uncertainties. In Table S3, the advancing and receding angles were determined directly from video frames at the moments when the contact line was advancing or receding, with standard deviations calculated from three repeated measurements. For the capillary bridge simulations, we used the receding contact angle observed from the corresponding capillary bridge retraction experiment. The values lie within the range of the experimentally measured CAH for the droplet setup (as reported in Table S3). This means the measurements remain sufficiently reliable for estimating the relevant contact angles, which is further supported by the good agreement between the experimental and simulated capillary bridge morphologies.

Table S3. Advancing and receding contact angle measurements in the surrounding liquid. All measurements were repeated at least three times to determine the standard deviation.

Surface	Droplet composition	Surrounding liquid	Advancing angle ± standard dev. (°)	Receding angle ± standard dev. (°)
Stainless Steel	Tetradecane	Water	118.2 ± 6.5	37.0 ± 7.1
Stainless Steel	Decane	Water	125.1 ± 6.0	53.4 ± 5.8
Stainless Steel	Tetradecane	Formulation	118.7 ± 3.9	30.8 ± 1.4
Glass	Decane	Water	132.6 ± 5.6	65.8 ± 1.3
Glass	Tetradecane	Water	132.2 ± 5.5	57.9 ± 1.4
Glass	Silicone oil	Water	129.2 ± 6.5	118.7 ± 4.9
Glass	Water (fluorescein dyed)	Tetradecane	81.2 ± 8.2	75.5 ± 11.3
PTFE	Tetradecane	Water	28.2 ± 3.4	13.0 ± 2.0
PTFE	Tetradecane	Formulation	32.0 ± 9.8	25.0 ± 4.8
PTFE	Decane	Water	26.0 ± 3.6	15.2 ± 2.0
PTFE	Decane	Formulation	65.1 ± 4.4	40.4 ± 2.8
PMMA	Water (fluorescein dyed)	Tetradecane	98.6 ± 8.9	88.5 ± 3.1
PMMA	Tetradecane	Formulation	106 ± 20.3	130.0 ± 4.6
PMMA	Tetradecane	Water	126.5 ± 7.0	55.6 ± 8.1
PMMA	Squalane	Water	119.6 ± 3.8	59.6 ± 6.0
PMMA	Olive oil	Water	113.0 ± 16.8	50.5 ± 6.0
PMMA	Decane	Water	89.1 ± 6.1	59.6 ± 4.4
PMMA	Tetradecane (Sudan IV dyed)	Water	81.1 ± 11.1	71.7 ± 12.2
PC	Tetradecane	Formulation	113.6 ± 9.4	50.2 ± 4.3
PC	Tetradecane	Water	73.9 ± 8.9	19.9 ± 3.7
PC	Decane	Water	78.0 ± 2.0	22.2 ± 5.1

AFM imaging was performed in contact mode using a JPK NanoWizard 3 (Bruker, USA) at ambient temperature and humidity. The AFM tip/cantilever (SNL-10 tip A, Bruker AFM Probes, USA, nominal spring constant 0.4 N m^{-1}) was calibrated using its thermal spectrum after the measurements. Representative AFM images and the corresponding roughness parameters (average ± standard deviation) were obtained from three repeated measurements across randomly selected areas of each sample. Roughness statistics were processed using Gwyddion[5] (Table S4).

Table S4. AFM topographic images of the substrates and corresponding roughness parameters. Each measurement was repeated at least three times to determine the standard deviation.

Material	AFM Topographic image	Mean roughness (Sa) in nm	RMS roughness (Sq) in nm	Surface area/projected area (r)
Stainless steel	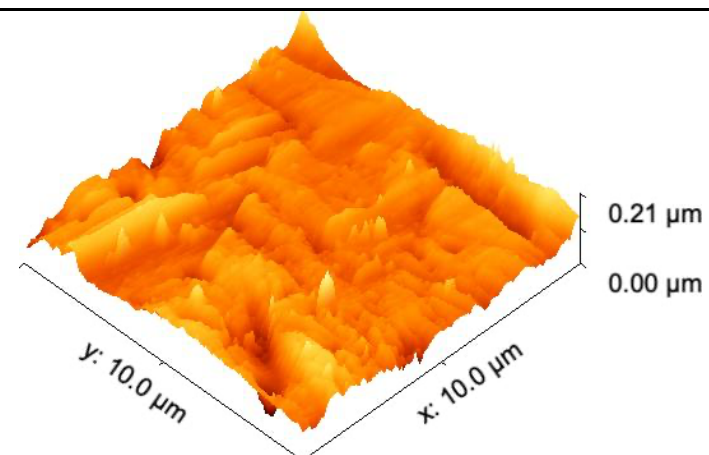	24.13 ± 13.38	33.37 ± 21.10	1.032 ± 0.030
Glass	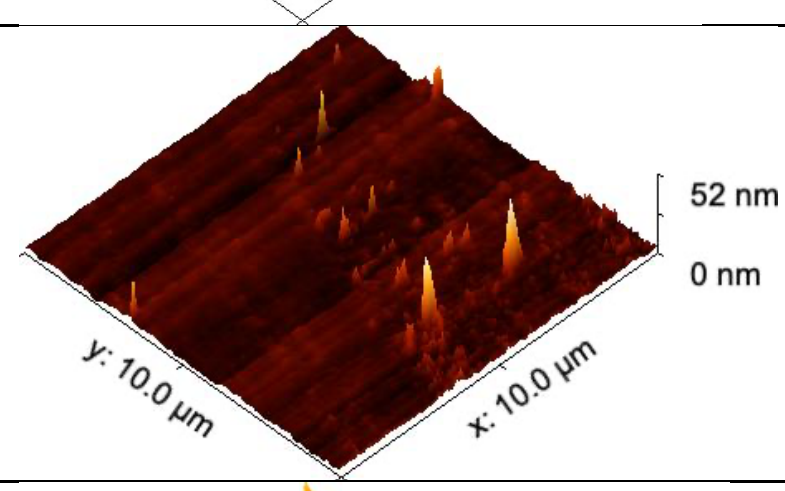	1.26 ± 0.39	1.71 ± 0.65	1.0004 ± 0.001
PMMA	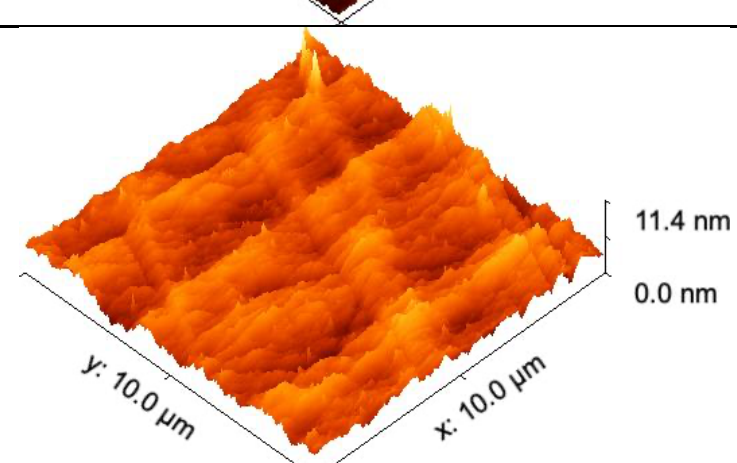	0.97 ± 0.18	1.62 ± 0.60	1.000 ± 0.001
PC	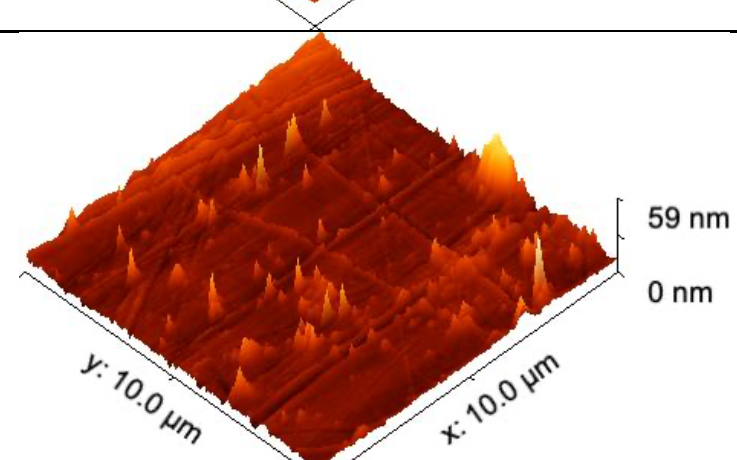	2.44 ± 0.58	4.21 ± 1.29	1.003 ± 0.002
PTFE	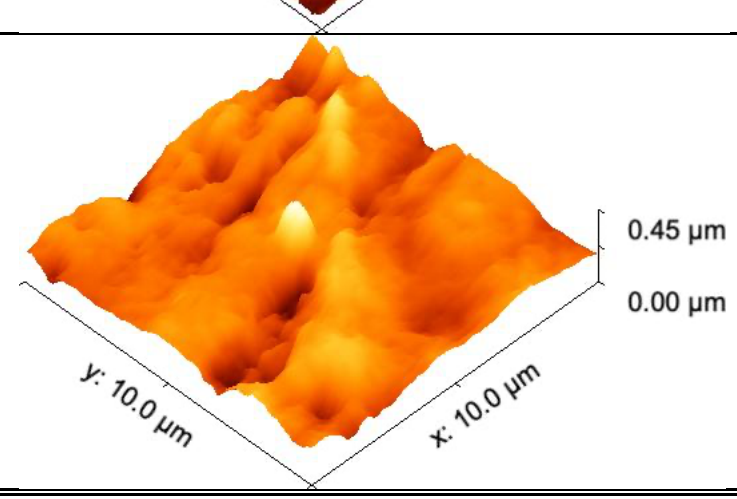	46.20 ± 10.14	63.87 ± 15.21	1.0296 ± 0.013

Supplementary Note 7: Impact of curved air interfaces on the regime diagram and partial dewetting examples

The theoretical line shown in the regime diagram in Fig. 3a of the main text corresponds to $\cos\theta_{dl} = \cos\theta_t$, where we have approximated $\cos\theta_t \approx (\gamma_{lg} - \gamma_{dg})/\gamma_{dl}$. This simple geometrical model assumes the liquid–air and droplet–air interfaces to be flat, while in practice these interfaces can be curved. Consequently, deviations can be observed close to the boundary line between dewetting and non-dewetting regimes. To illustrate this, Fig. S6 examines the transition region in detail for fixed interfacial systems with varying θ_{dl}. The red dashed lines indicate contact angles satisfying $\cos\theta_{dl} = \cos\theta_t$, while the black dash lines correspond to 50% dewetting. Small deviations of <10º are typical, which explain the good agreements between experiments, simulations, and the simple model in Fig. 3 of the main text.

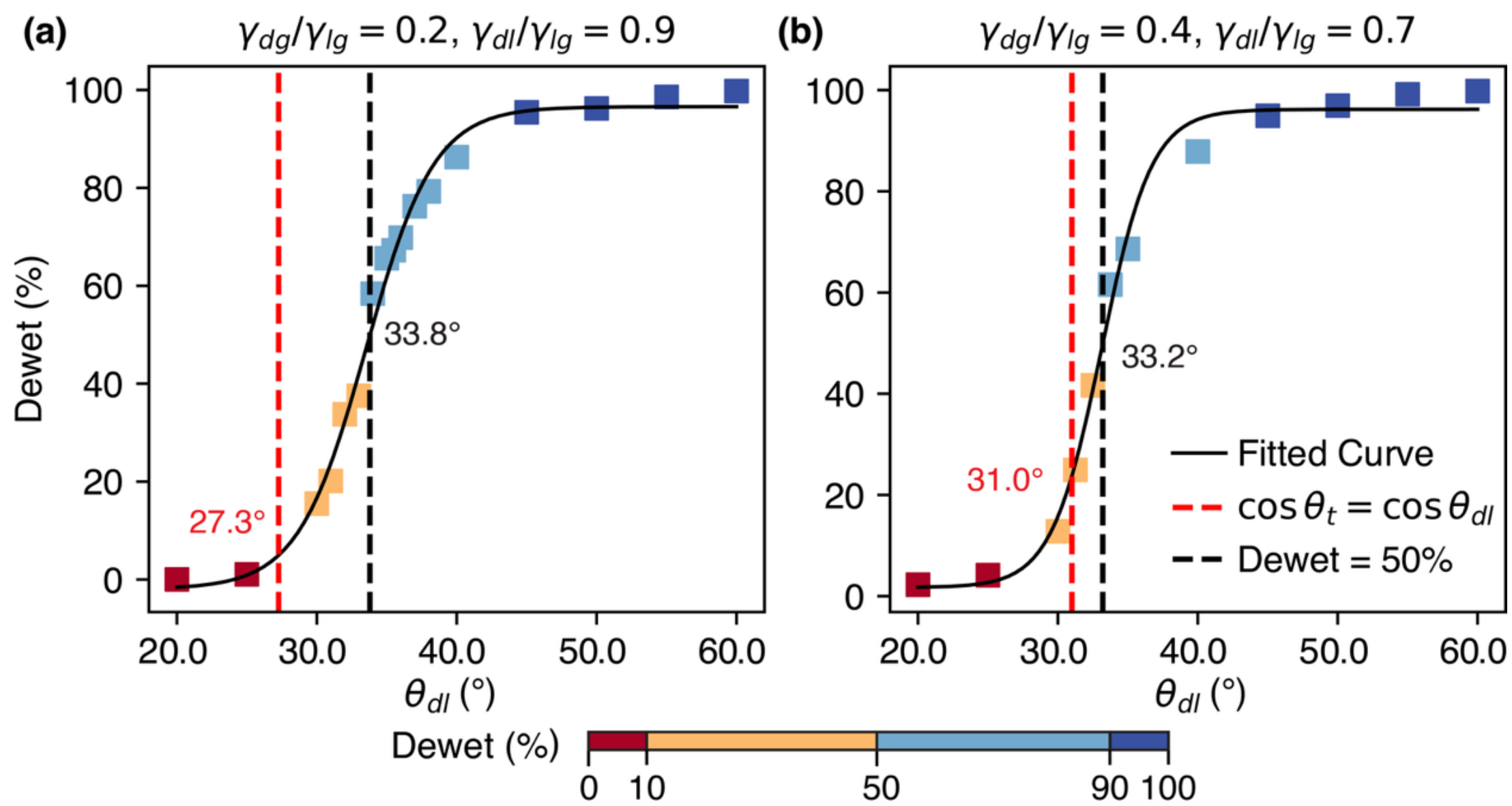

Figure S5. Dewetting and non-dewetting boundaries for fixed interfacial systems. (a) $\gamma_{dg}/\gamma_{lg} = 0.2$, $\gamma_{dl}/\gamma_{lg} = 0.9$; and (b) $\gamma_{dg}/\gamma_{lg}=0.4$; $\gamma_{dl}/\gamma_{lg}=0.7$. The bottom effective contact angle θ_{dl} is varied, and the data points are fitted with a hyperbolic tangent function. The theoretical boundary $\cos\theta_{dl} = \cos\theta_t$ is shown as a red dashed line, while the simulation predicted 50% dewetting threshold is indicated by a black dashed line.

Here we show example partial dewetting simulation snapshots illustrating the configuration evolution of the capillary bridge as it stretches and ruptures, ultimately leading to the separation of the droplet into two individual bodies.

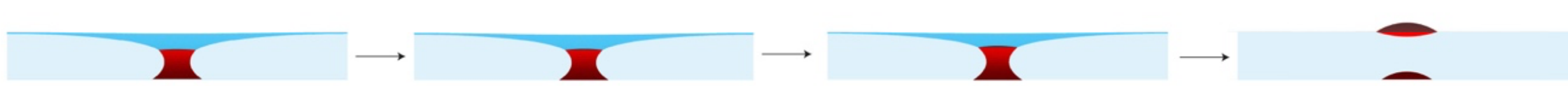

Figure S6. Example of simulation results showing partial dewetting. The system parameters are $\gamma_{dg}/\gamma_{lg}=0.4$; $\gamma_{dl}/\gamma_{lg}=0.7$; $\theta_{dl}=35.0°$. From left to right, the working liquid rises.

Supplementary Note 8: Experimental dewetting outcome compared with simulation prediction

Dewetting experiments were performed across a range of fluids and substrates, with each experiment repeated at least 3 times to obtain standard deviations. While most experiments are consistent with simulations, slight deviations are observed (Table S5). This discrepancy arises from the fact that simulations exhibit nearly binary behaviour, e.g. either complete dewetting or non-dewetting, with a sharp transition in between (see Fig. 3 of the main text). In contrast, experimental outcomes can be affected by substrate heterogeneity, surface roughness, and dynamic effects during the final pinch-off of the capillary bridge, all of which contribute to deviations from the quasi-static simulation predictions on perfectly smooth and homogenous surfaces.

Table S5. Measured dewetting results and simulation prediction.

Droplet	Working liquid	Substrate	Experiment dewet ± standard deviation (%)	Simulation predicted dewet (%)
Decane	Water	PTFE	4 ± 2	0
Decane	Water	Stainless steel	82 ± 8	100
Decane	Water	PC	62 ± 4	100
Decane	Water	PMMA	86 ± 4	100
Decane	Water	Glass	74 ± 3	100
Tetradecane	Formulation	PTFE	79 ± 3	100
Tetradecane	Formulation	Stainless steel	96 ± 1	100
Tetradecane	Formulation	PC	94 ± 8	100
Tetradecane	Formulation	PMMA	96 ± 1	100
Tetradecane	Water	PMMA	92± 1	100
Tetradecane	Water	Glass	97 ± 2	100
Tetradecane (Sudan IV dyed)	Water	PMMA	93 ± 2	100
Silicone oil	Water	Glass	88 ± 6	100
Squalane	Water	PMMA	91 ± 5	100
Olive oil	Water	PMMA	79 ± 5	100
Water (fluorescein dyed)	Tetradecane	PMMA	0 ± 0	0
Water (fluorescein dyed)	Tetradecane	Glass	0 ± 0	0

Supplementary Note 9: Interfacial measurements in binary and ternary system

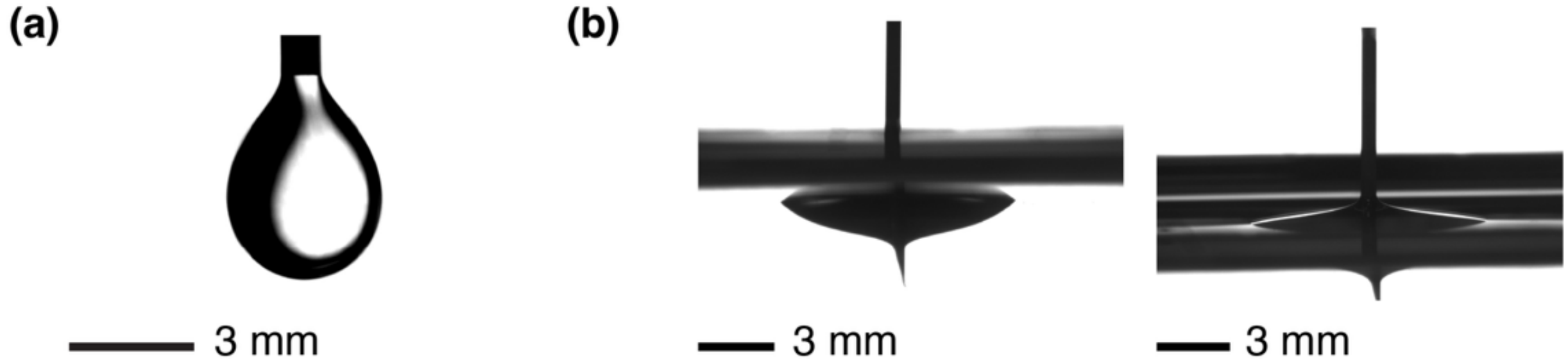

Figure S7. Interfacial tension measurements in binary and ternary systems. Binary systems were measured using the pendant drop method, and ternary systems using the Neumann triangle method. (a) Pendant drop measurement of a tetradecane droplet suspended in the formulated working fluid. (b) Neumann triangle analysis of a tetradecane droplet floating as a liquid lens at the interface between the formulated working fluid and air.

Supplementary Note 10: Hansen distance calculation

To evaluate the solubility of the between components in this multiphase system where glycol ethers formulated in the working fluid, we employed Hansen solubility parameter (HSP) analysis to assess the likelihood of glycol ether migration across the oil–water interface. The interaction distances $R_{a(A-B)}$ of two molecules A and B in Hansen space is calculated using Eq. S1:

$$R_{a(A-B)} = \sqrt{4(\delta_{d,A} - \delta_{d,B})^2 + (\delta_{p,A} - \delta_{p,B})^2 + (\delta_{h,A} - \delta_{h,B})^2} \qquad \text{(S1)}$$

where δ_d, δ_p, and δ_h are contributions to the total cohesive energy density from dispersion forces, dipole–dipole interactions, and hydrogen bonding, respectively.

Using the data from Table S6, the calculated Hansen distance $Ra_{(A-B)}$ between the glycol ethers and the oil phase (decane or tetradecane) range from 10.5 to 15.3 (J $cm^{-3})^{1/2}$, with an average of 12.66 (J $cm^{-3})^{1/2}$. In contrast, the distance to water is significantly larger, ranging from 30.9 to 34.9(J $cm^{-3})^{1/2}$, with an average of 33.03 (J $cm^{-3})^{1/2}$. This approximately 2.6 times lower Hansen distance to the oil indicates a much higher solubility of the glycol ethers in the oil phase, suggesting that they preferentially migrate toward the soil droplet.

Table S6. Hansen solubility parameters.

	HSP ([J $cm^{-3}]^{1/2}$)		
	δ_d	δ_p	δ_h
Diethylene glycol monoethyl ether (Carbitol ™)	16.1[a]	9.2[a]	12.2[a]
Tripropylene glycol methyl ether (DOWANOL™ TPM)	15.1[b]	3.5[b]	11.5[b]
Ethylene glycol monohexyl ether (Hexyl CELLOSOLVE™)	16.2[c]	9c	5.5[c]
Decane	15.7[d]	0[d]	0[d]
Tetradecane	16.2[d]	0[d]	0[d]
Water	15.5[d]	16[d]	42.3[d]

[a] from Dow database, Technical Data Sheet, https://www.dow.com/en-us/document-viewer.html?docPath=/content/dam/dcc/documents/110/110-00978-01-carbitol-solvent-tds.pdf
[b] from Dow database, Technical Data Sheet, https://www.dow.com/en-us/document-viewer.html?docPath=/content/dam/dcc/documents/110/110-00619-01-dowanol-tpm-tds.pdf
[c] from Dow database, Technical Data Sheet, https://www.dow.com/en-us/document-viewer.html?docPath=/content/dam/dcc/documents/110/110-00971-01-hexyl-cellosolve-solvent-tds.pdf
[d] taken from Ref. [4].

Supplementary Note 11: Soaking experiments for systems shown in main text

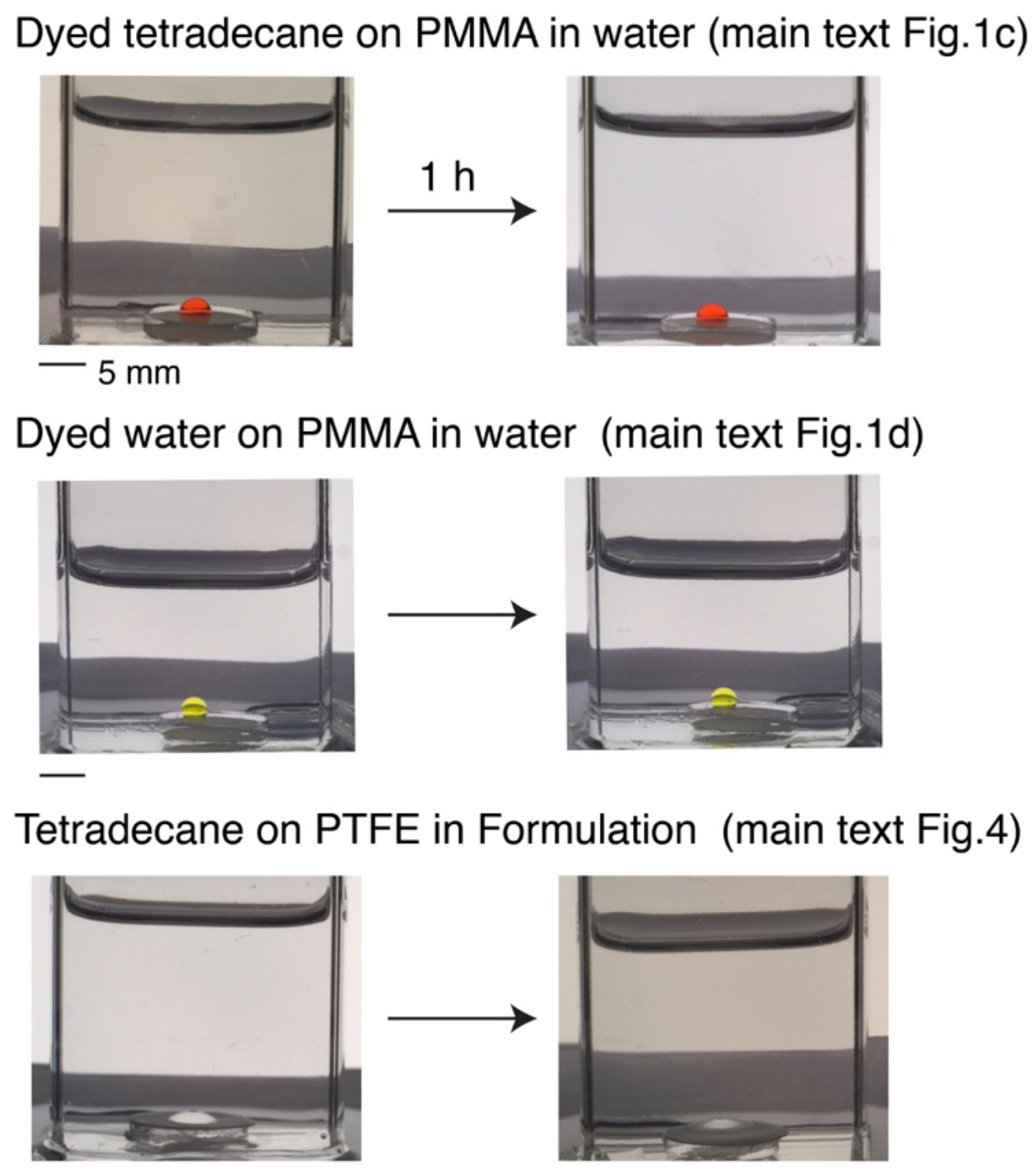

Figure S8. Droplet soaking experiments for the systems shown in Fig. 1 and Fig. 4 of the main text. A 10 µL olive-oil droplet was deposited on the corresponding substrate at the bottom of a bath of the surrounding fluid. The system was left undisturbed for 1 h, and the droplet remained in place, showing no detachment.

Supplementary Note 12: Soaking experiments with added detergent

The soaking experiment in Fig. 5 of the main text uses pure water as the working fluid. However, in real-world applications, it is common to add washing detergents to the water bath to aid soil removal. To evaluate this scenario, we added 0.1 mL of a 50 w/w% solution of Fairy washing-up liquid to a 10 mL water bath containing a 10 µL olive oil droplet representing the soil, see Fig. S9. We observe that the detergent took approximately 40 minutes to reach the droplet and an additional 10 minutes to remove it, typically in two or more stages. This process is significantly slower than the capillary lifting strategy presented in this work, where around 80% of the droplet is lifted within seconds without surfactant input.

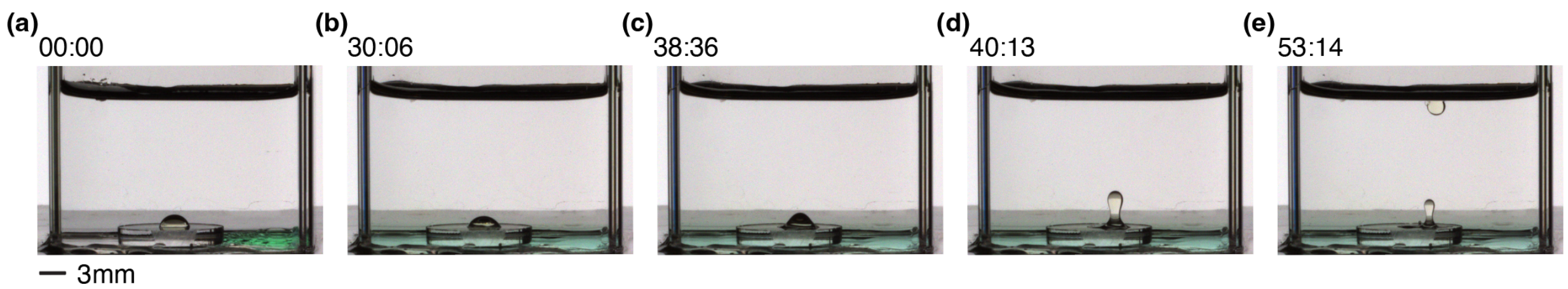

Figure S9. Droplet removal by soaking with a solution containing detergent. (a) A 0.1 mL solution of 50 w/w% Fairy in water is added to a 10 mL water bath containing a 10 µL olive oil droplet deposited on a PMMA substrate at the bottom. (b) Within the first 30 minutes, the washing-up liquid diffuses around the bottom of the container, lowering the water-droplet interfacial tension and causing the droplet to spread. (c) Then, the washing-up liquid deforms the upper curvature of the droplet, initiating detachment from the central top. This process continues until a significant portion of the droplet is lifted and detached, as shown in (d). After some time, the effect repeats, detaching a second portion of the droplet (e). Timestamps are shown in mm:ss.

Supplementary References

1. Daerr, A. & Mogne, A. Pendent_Drop: An ImageJ Plugin to Measure the Surface Tension from an Image of a Pendent Drop. *J. Open Res. Softw.* **4**, e3 (2016).
2. Schneider, C. A., Rasband, W. S. & Eliceiri, K. W. NIH Image to ImageJ: 25 years of image analysis. *Nat. Methods* **9**, 671–675 (2012).
3. Stalder, A. F. *et al.* Low-bond axisymmetric drop shape analysis for surface tension and contact angle measurements of sessile drops. *Colloids and Surfaces A: Physicochemical and Engineering Aspects* **364**, 72–81 (2010).
4. Murase, M. & Nakamura, D. Hansen Solubility Parameters for Directly Dealing with Surface and Interfacial Phenomena. *Langmuir* **39**, 10475–10484 (2023).
5. Nečas, D. & Klapetek, P. Gwyddion: an open-source software for SPM data analysis. *Cent. Eur. J. Phys.* **10**, 181–188 (2012).
